# Remasking of Candida albicans β-Glucan in Response to Environmental pH Is Regulated by Quorum Sensing

**DOI:** 10.1128/mBio.02347-19

**Published:** 2019-10-15

**Authors:** Fabien Cottier, Sarah Sherrington, Sarah Cockerill, Valentina del Olmo Toledo, Stephen Kissane, Helene Tournu, Luisa Orsini, Glen E. Palmer, J. Christian Pérez, Rebecca A. Hall

**Affiliations:** a Institute of Microbiology and Infection, School of Biosciences, University of Birmingham, Birmingham, United Kingdom; b Interdisciplinary Center for Clinical Research, University Hospital Würzburg, Würzburg, Germany; c Institute for Molecular Infection Biology, University of Würzburg, Würzburg, Germany; d Environmental Genomics Group, School of Biosciences, University of Birmingham, Birmingham, United Kingdom; e Department of Hematology and Oncology, College of Medicine, University of Tennessee Health Sciences Center, Memphis, Tennessee, USA; f Department of Clinical Pharmacy and Translational Science, College of Pharmacy, University of Tennessee Health Sciences Center, Memphis, Tennessee, USA; Duke University Medical Center

**Keywords:** *Candida albicans*, cell wall, chitin, glucans, pH sensing, quorum sensing

## Abstract

Candida albicans is a commensal yeast of the human gut which is tolerated by the immune system but has the potential to become an opportunistic pathogen. One way in which C. albicans achieves this duality is through concealing or exposing cell wall pathogen-associated molecular patterns (PAMPs) in response to host-derived environment cues (pH, hypoxia, and lactate). This cell wall remodeling allows C. albicans to evade or hyperactivate the host’s innate immune responses, leading to disease. Previously, we showed that adaptation of C. albicans to acidic environments, conditions encountered during colonization of the female reproductive tract, induces significant cell wall remodeling resulting in the exposure of two key fungal PAMPs (β-glucan and chitin). Here, we report that this pH-dependent cell wall remodeling is time dependent, with the initial change in pH driving cell wall unmasking, which is then remasked at later time points. Remasking of β-glucan was mediated via the cell density-dependent fungal quorum sensing molecule farnesol, while chitin remasking was mediated via a small, heat-stable, nonproteinaceous secreted molecule(s). Transcript profiling identified a core set of 42 genes significantly regulated by pH over time and identified the transcription factor Efg1 as a regulator of chitin exposure through regulation of *CHT2*. This dynamic cell wall remodeling influenced innate immune recognition of C. albicans, suggesting that during infection, C. albicans can manipulate the host innate immune responses.

## Abstract (precis)

Candida albicans is part of the microbiota of the skin and gastrointestinal and reproductive tracts of humans and has coevolved with us for millennia. During that period, C. albicans has developed strategies to modulate the host’s innate immune responses, by regulating the exposure of key epitopes on the fungal cell surface. Here, we report that exposing C. albicans to an acidic environment, similar to the one of the stomach or vagina, increases the detection of the yeast by macrophages. However, this effect is transitory, as C. albicans is able to remask these epitopes (glucan and chitin). We found that glucan remasking is controlled by the production of farnesol, a molecule secreted by C. albicans in response to high cell densities. However, chitin-remasking mechanisms remain to be identified. By understanding the relationship between environmental sensing and modulation of the host-pathogen interaction, new opportunities for the development of innovative antifungal strategies are possible.

## INTRODUCTION

Recognition is a critical step for any immune response to be triggered. Initial protection from infection is largely mediated via the innate immune system composed of dendritic cells, macrophages, and polymorphonuclear neutrophils (1). These cells recognize exposed epitopes on the surfaces of invading pathogens and trigger the first immune response and are essential for the development of adaptive immunity. However, the human body hosts trillions of viruses, bacteria, and fungi without constant activation of the immune system (2, 3). This is the result of millennia of coevolution between humans and their microbiome, where our immune system developed several receptors and mechanisms to identify and eliminate pathogens and to tolerate the presence of commensal organisms. This fine-tuning of innate immune responses prevents excessive inflammation while protecting the host (4). However, many pathogens are capable of hijacking the innate immune system to evade detection and to promote their dissemination (5, 6).

Candida albicans forms part of the natural human microbiota, colonizing the human body as early as the first month after birth (7), and can end up representing 0.01% of the gut microbiota (8). In addition to forming part of the gut microbiome, C. albicans is also routinely found on the skin and the oral and vaginal mucosa (9). However, during periods of dysbiosis or immune suppression, this commensal organism can proliferate and cause a large variety of diseases, ranging from superficial thrush to life-threatening systemic bloodstream infections associated with high mortality rates (10, 11).

C. albicans virulence is predominantly supported by its ability to switch between yeast and hyphal growth (12). This morphological transition is regulated by several environmental cues, including pH, temperature, hypercapnia, hypoxia, carbon source, *N*-acetylglucosamine, serum, or contact with solid surfaces (13). Both morphologies are present in biofilms, structures providing antifungal resistance to C. albicans, where yeast promote dissemination, while hyphae are critical for surface colonization, tissue penetration, and the production of the toxin candidalysin (14). C. albicans exists in four different yeast morphologies (white, opaque, gray, and gut), which are specialized for colonizing specific host niches (15). Each of these morphotypes can be visually identified but are also associated with specific transcription profiles and fundamental reorganization of the fungal cell wall (16, 17).

The fungal cell wall is a highly dynamic organelle which plays a pivotal role in the host-pathogen interaction. In C. albicans, the cell wall is composed of an inner layer of chitin and β-glucan and an external layer of heavily glycosylated mannoproteins (18). All of these cell wall carbohydrates act as pathogen-associated molecular patterns (PAMPs), which are recognized by pattern recognition receptors (PRRs) expressed on the surfaces of innate immune cells. For example, dectin-1 recognizes β-1,3-glucan (19), while Toll-like receptor 4 (TLR4) (20), the mannose receptor (21), dectin-2, and mincle recognize the mannoproteins to elicit a strong proinflammatory innate immune response (22, 23). However, recognition of fungal chitin through NOD2 results in the production of interleukin 10 (IL-10), inducing fungal tolerance (24, 25). Therefore, depending on the PAMPs exposed on the C. albicans surface, the host immune system triggers various responses that the yeast can exploit to improve its survivability.

C. albicans has evolved multiple pathways to control the exposure of cell surface epitopes, allowing the fungus to manipulate the host’s innate immune responses. Particularly, the fungal cell wall is highly remodeled during morphogenesis and upon adaptation to key host-derived environmental signals (18). For example, in hyphal cells, the β-1,3-glucan is largely concealed from the innate immune system by the outer mannoprotein layer (26), but can become exposed during infection and induce proinflammatory immune responses, due to the action of neutrophils (27). β-1,3-Glucan and chitin are also exposed during adaptation to acidic environments, a key environmental signal of the female reproductive tract, driving enhanced neutrophil recruitment and secretion of proinflammatory cytokines (28). On the other hand, growth in lactate or under hypoxic conditions induces masking of β-glucan, resulting in evasion of innate immune responses (29–31). Despite both environmental signals inducing β-glucan masking, these signals are mediated via different mechanisms, with lactate sensing involving the Gpr1 receptor and the Crz1 and Ace2 transcription factors and hypoxia-induced β-glucan masking mediated via the cAMP-protein kinase A (PKA) pathway.

During infection, PAMP exposure of C. albicans continuously changes, making the host-pathogen interaction highly variable (32). Here, we investigated the dynamics of PAMP exposure in response to growth in an acidic environment, akin to conditions encountered at the vaginal mucosa. Upon initial adaptation to acidic environments, C. albicans unmasked its β-glucan and chitin, with peak exposure occurring between 2 and 4 h of growth. However, the cell wall then underwent a second phase of cell wall remodeling, resulting in the remasking of these PAMPs. This remasking was mediated via the secretion of the fungal quorum sensing molecule farnesol and an unidentified small, heat-stable, nonproteinaceous secreted molecule(s). RNA-sequencing analysis identified a core group of 42 genes regulated between pH and through time and identified Efg1 has a regulator of chitin exposure in response to environmental pH.

## RESULTS

### Unmasking of fungal PAMPs in response to environmental pH is time dependent.

**FIG 1:**
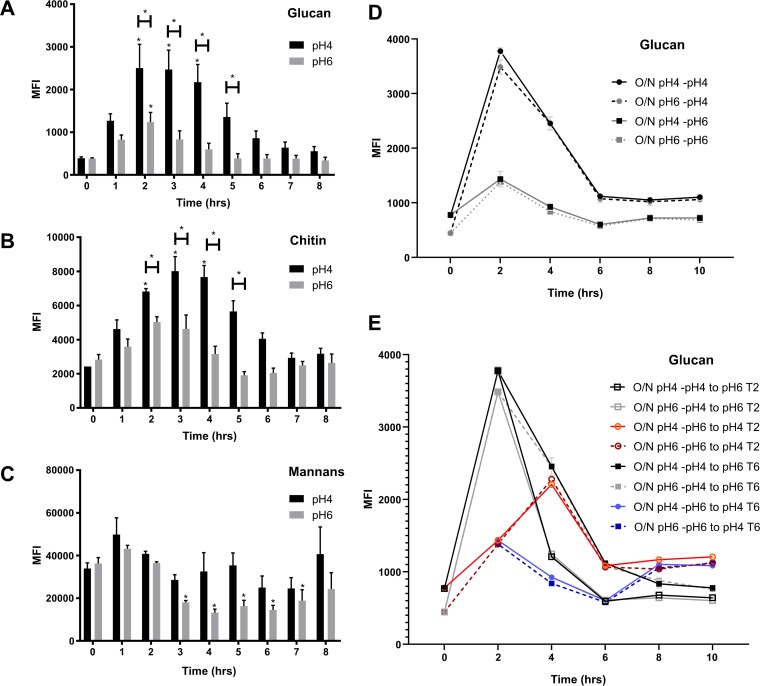
Glucan and chitin exposure is dependent on time and environmental pH. Wild-type strain (SC5314) was grown in fresh YPD buffered at pH 4 or pH 6 at 37°C for 8 h to 10 h. Cells were fixed and stained with FITC or TRITC label, and median fluorescence intensity of exposed β-1,3-glucan (A, D, E), exposed chitin (B), and total mannans (C) were quantified by FACS. (D) SC5314 cells were grown overnight in pH 4 or pH 6 medium and then freshly inoculated in YPD medium at pH 4 or 6 and incubated at 37°C. (E) Cultures were then swapped to different fresh medium 2 and 6 h postinoculation. Data represent the means and SEMs from seven biological replicates and analyzed by 2-way ANOVAs with Sidak’s *post hoc* tests. *, *P* < 0.05 versus respective control at *T*_0_.

Previously, we showed that C. albicans actively remodels its cell wall during adaptation to environmental pH (28). To investigate the dynamics of this cell wall remodeling, we analyzed carbohydrate exposure in response to environmental pH over time. Standard yeast extract-peptone-dextrose (YPD) overnight cultures (pH ∼6) showed minimal PAMP (chitin and β-glucan) exposure (*T*_0_). In agreement with our previous data (28), we identified a significant increase of carbohydrate exposure when cells were subcultured in fresh YPD buffered at pH 4 compared to those in YPD buffered at pH 6 after 4 h of growth (Fig. 1A and B). This unmasking of the cell wall was a rapid process, with significant PAMP exposure occurring within 2 h from inoculation in fresh medium. However, this cell wall remodeling was a transient process, and the cell wall underwent significant remasking of these PAMPs at later time points, despite the pH of the medium remaining constant throughout the time course. The unmasking and remasking of glucan and chitin did not correlate with significant changes in mannan content, as determined by concanavalin A (ConA) staining (Fig. 1C), suggesting that the total amount of mannan in the cell wall does not regulate the exposure of these important PAMPs. However, ConA is a nonspecific stain providing information on the total amount of mannan but not the specific structure. Therefore, during adaptation to acidic environments, C. albicans undergoes both unmasking and remasking of fungal cell wall PAMPs that are critical for innate immune recognition.

To determine whether the conditions of the overnight culture were important for cell wall remodeling to occur, we grew C. albicans overnight in pH 4 or pH 6 medium and then reinoculated each overnight culture in fresh pH 4 and pH 6 media. Cultures grown overnight at either pH 4 or pH 6 had similar carbohydrate exposure levels at *T*_0_. Independent of the pH of the overnight culture, inoculation in fresh pH 4 medium resulted in glucan and chitin exposure 2 h postinfection, followed by a remasking of both PAMPs 6 h postinoculation (Fig. 1D; see also Fig. S1C in the supplemental material). To determine whether the time of inoculation is important for cell wall remodeling to occur, the pH of the medium was switched 2 h and 6 h postinoculation. Independent of the time of inoculation, switching C. albicans to acidic medium resulted in elevated PAMP exposure, while switching the medium to pH 6 resulted in rapid PAMP remasking (Fig. 1E and S1D). Therefore, the observed cell wall remodeling was specific to a change in environmental pH and occurred independently of the growth phase.

10.1128/mBio.02347-19.1 FIG S1 Remasking of fungal cell wall PAMPs requires metabolically active cells. Wild-type C. albicans (SC5314) was grown in fresh YPD buffered at pH 4 or pH 6 at 37°C for 2 h, and then cultures were either maintained at 37°C or at 4°C. Cells were fixed and stained for glucan (A) and chitin (B, C, D) exposure. (C) SC5314 cells were grown overnight in pH 4 or pH 6 medium and then freshly inoculated in YPD medium at pH 4 or 6 and incubated at 37°C. (D) Cultures were then swapped to different fresh medium 2 and 6 h postinoculation. Data represent the means and SEMs from three biological replicates and analyzed by 2-way ANOVA with Sidak’s *post hoc* tests. *, *P* < 0.05. Download FIG S1, TIF file, 1.2 MB. Copyright © 2019 Cottier et al. 2019 Cottier et al. This content is distributed under the terms of the Creative Commons Attribution 4.0 International license.

To determine whether remasking of these cell wall PAMPs is mediated via an active mechanism, C. albicans cells from 2 h cultures were resuspended in fresh buffered medium cooled to 4°C and were incubated at 4°C for an additional 7 h. Contrary to those at 37°C, C. albicans cells maintained at 4°C in pH 4 buffered YPD did not undergo remasking of either β-glucan or chitin (Fig. S1A and B), with exposure levels remaining comparable to those at the initial 2 h time point. Therefore, remasking of the cell wall in response to acidic pH is an active process requiring the cells to be metabolically active.

### Time-dependent pH exposure of fungal cell wall PAMPs occurs in clinical C. albicans strains and other dimorphic *Candida* species.

**FIG 2:**
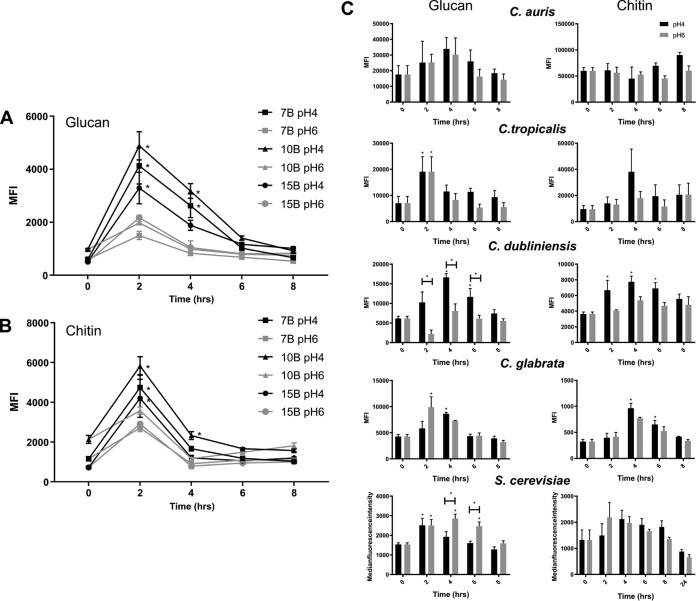
pH-dependent cell wall remodeling is associated with dimorphic *Candida* species. Clinical strains were grown in fresh YPD buffered at pH 4 or pH 6 at 37°C for 8 h. Cells were fixed and stained with FITC or TRITC label, and the median fluorescence intensities of exposed β1,3-glucan (A) and exposed chitin (B) were quantified by FACS. (C) Non-*albicans Candida* species were stained for glucan (left) and chitin (right) exposure over time. Data represent the means and SEMs from three biological replicates and analyzed by 2-way ANOVAs with Sidak’s *post hoc* tests. *, *P* < 0.05.

To confirm that the regulation of PAMP exposure by pH is not restricted to laboratory strains of C. albicans, we investigated pH-dependent PAMP exposure in several clinical C. albicans isolates (7B, 10B, and 15B). In agreement with our previous results, these clinical isolates initially underwent β-glucan and chitin unmasking with peak exposures between 2 and 4 h, followed by significant cell wall remasking from 6 h onwards (Fig. 2A and B). Therefore, dynamic cell wall remodeling in response to environmental pH is a general trait of C. albicans.

To identify whether this regulation of PAMP exposure is specific to C. albicans, chitin and β-glucan exposure in response to environmental pH was quantified in non-*albicans Candida* species and Saccharomyces cerevisiae. Candida dubliniensis exhibited similar kinetics in glucan exposure as C. albicans, displaying initial unmasking and the subsequent remasking of glucan when grown under acidic conditions (Fig. 2C). However, unlike C. albicans, C. dubliniensis did not show significant pH-dependent chitin exposure, although there was a trend that chitin exposure was increased under acidic growth conditions, which declined over time (Fig. 2C). The cell wall of Candida tropicalis showed fluctuations in PAMP exposure, but these were not pH dependent (Fig. 2C). S. cerevisiae, on the other hand, showed an inverse regulation of glucan exposure, with glucan being masked at low pH. This glucan masking was also time dependent, as exposed glucan levels were comparable between cells grown at pH 4 and those grown at pH 6 after 8 h of growth (Fig. 2C). Neither of the nondimorphic yeasts, Candida glabrata and Candida auris, showed pH- or time-dependent cell wall remodeling (Fig. 2C). Therefore, pH-dependent cell wall remodeling appears to be a trait of dimorphic *Candida* species, although whether dimorphism and cell wall remodeling are directly linked remains to be investigated.

### PAMP exposure influences macrophage phagocytosis rates, intracellular survival, and virulence.

**FIG 3:**
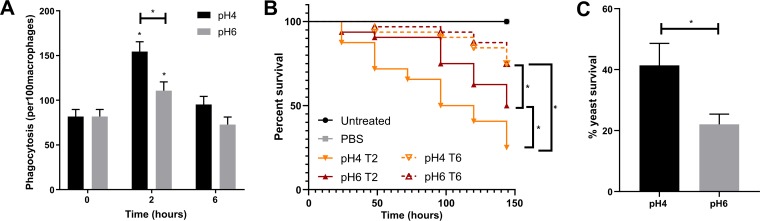
PAMP exposure influences macrophage phagocytosis, intracellular survival, and virulence. Wild-type C. albicans (SC5314) was grown in fresh YPD buffered at pH 4 or pH 6 at 37°C for 2 or 6 h. (A) Cells were then coincubated with J774.1A macrophages at an MOI of 5 for 1 h, and the rate of phagocytosis determined. (B) Cumulative survival curve of G. mellonella larvae infected with 2 × 10^5^ cells. Experiment performed in 3 biological replicates with 10 larvae per group each time. Comparisons were performed by log rank (Manter-Cox) test. (C) C. albicans survival percentage after 3 h of incubation with human neutrophils. Data represent the means and SEMs from six individual experiments and analyzed by *t* tests. *, *P* < 0.05.

Previously, variations in C. albicans glucan exposure were correlated with phagocytosis rates of macrophages and neutrophils (28, 29, 33). To validate this observation in our system, we exposed J774.1A macrophages to C. albicans cells grown in YPD buffered at pH 4 or pH 6 for 2 and 6 h. As expected, phagocytosis rates were significantly increased at early time points when β-glucan exposure was high, with acid-adapted cells displaying the highest phagocytosis rates. However, phagocytosis rates significantly decreased upon the initiation of cell wall remasking (Fig. 3A). These results validate the relationship between C. albicans PAMP exposure and phagocytosis by macrophages.

To investigate the potential impact of this pH-dependent fluctuation in PAMP exposure on pathogenicity, we infected Galleria mellonella with 2 × 10^5^ C. albicans cells that had been grown at pH 4 or pH 6 for 2 and 6 h and monitored larval survival over 6 days. Interestingly, C. albicans cells grown at pH 4 for 2 h displayed a significantly higher virulence than cells grown at pH 6 (Fig. 3B). Furthermore, C. albicans cells grown at either pH 4 or pH 6 for 6 h were significantly less virulent than cells grown for 2 h. Therefore, the increased level of PAMP exposure in actively growing cells correlates with higher pathogenicity.

Given that pH-dependent PAMP exposure results in elevated phagocytosis, but increased virulence, we assessed the ability of key phagocytes to kill C. albicans adapted to either pH 4 or pH 6. C. albicans cells grown at pH 4 for 4 h survived significantly longer inside human neutrophils than cells grown at pH 6 (Fig. 3C). Therefore, acid-adapted C. albicans exhibits higher phagocytosis rates but survives within the phagocytes and is associated with enhanced virulence.

### Remasking of fungal PAMPs is not linked to intracellular pH recovery or to intracellular superoxide level.

Recently, growth at acidic pH has been shown to reduce cytoplasmic pH and modulate cAMP signaling through activation of Cyr1 (34), and activation of the cAMP pathway has been linked to glucan masking in response to other environmental stimuli. Therefore, we hypothesized that pH-dependent cell wall remasking may relate to recovery of intracellular pH over time and to time-dependent modulation of cAMP signaling. Quantification of the intracellular pH (pH_i_) suggested that, under our conditions, there was no significant difference in pH_i_ values between C. albicans cells grown at pH 4 and those grown at pH 6 (Fig. S2A). However, the pH_i_ did change over time, but there was no significant difference between cells grown at pH 4 and at pH 6. Therefore, the effects of external pH on pH_i_ and, subsequently, on cell wall organization are not significant.

10.1128/mBio.02347-19.2 FIG S2 pH-induced cell wall remodeling is not regulated by changes in intracellular pH or cAMP signaling. (A) Growth and intracellular pH were measured in CAI4+pKE4-PHL2 cells grown in YNB at 37°C for 48 h. (B) Cells were incubated with a superoxide dye, and cellular fluorescence was measured by microscopy. C. albicans controls (SN250 and CAI4+pSM2) and mutant strains were inoculated in fresh YPD buffered at pH 4 or pH 6 and grown at 37°C for 3 and 7 h. Cells were then fixed and stained for glucan (C) and chitin (D) exposure. Data represent the means and SEMs from three biological replicates and analysed by 2-way ANOVA with Sidak’s *post hoc* tests. *, *P* < 0.05. Download FIG S2, TIF file, 1.4 MB. Copyright © 2019 Cottier et al. 2019 Cottier et al. This content is distributed under the terms of the Creative Commons Attribution 4.0 International license.

Glucan masking in response to hypoxia is mediated via reactive oxygen species (ROS) directly through activation of the cAMP-PKA pathway (30). As cells age, ROS accumulates inside cells. Therefore, elevated ROS levels in older cells may activate the cAMP-PKA signaling pathway leading to cell wall masking. To explore the role of ROS in pH-dependent cell wall remodeling, we used a fluorescent reporter to measure superoxide levels in C. albicans cells grown in YPD buffered at pH 4 or pH 6. In agreement with Pradhan et al. (30), we observed a significant reduction in superoxide levels in C. albicans cells at early time points when glucan exposure was at its maximum, with superoxide levels significantly lower at pH 4 than at pH 6 (Fig. S2B). As expected, this pH-dependent difference in superoxide levels was absent at later time points (*T*_6_) once cell wall remasking was initiated. However, superoxide levels were significantly lower at the later time points than at initial exposure (2 h). Given that β-glucan masking has been associated with high intracellular ROS, it was expected that superoxide levels would be significantly increased at the later time points. Therefore, in terms of pH-dependent cell wall remodeling, ROS levels are correlative rather than predictive of β-glucan exposure.

ROS are predicted to activate the cAMP-PKA pathway to regulate glucan exposure in response to hypoxia (30). This mechanism is controlled by *CYR1*, *TPK1*, and *TPK2*. We measured the level of β-glucan and chitin exposure in response to environmental pH in mutants of these genes. As previously reported, single *tpk1* or *tpk2* mutations did not induce alteration in PAMP exposure compared to the control strain. However, the *cyr1* as well as double *tpk1/tpk2* mutants showed no pH-dependent PAMP exposure, with both mutants displaying constitutive PAMP exposure (Fig. S2C), suggesting that the cAMP-PKA pathway regulates pH-dependent cell wall remodeling. However, both mutants had significant growth defects, with the *cyr1* and *tpk1/tpk2* double mutants having doubling times of 2.5 and 3.5 h, respectively, compared to the *tpk1* and *tkp2* mutants and control strain doubling in ∼1 h. Reduced fitness has been linked to many nonspecific phenotypes in C. albicans, including alterations in the cell surface (35). Therefore, from these experiments, it is impossible to disentangle the effects of pH from the reduced fitness.

### Secreted factors regulate remasking of fungal PAMPs.

**FIG 4:**
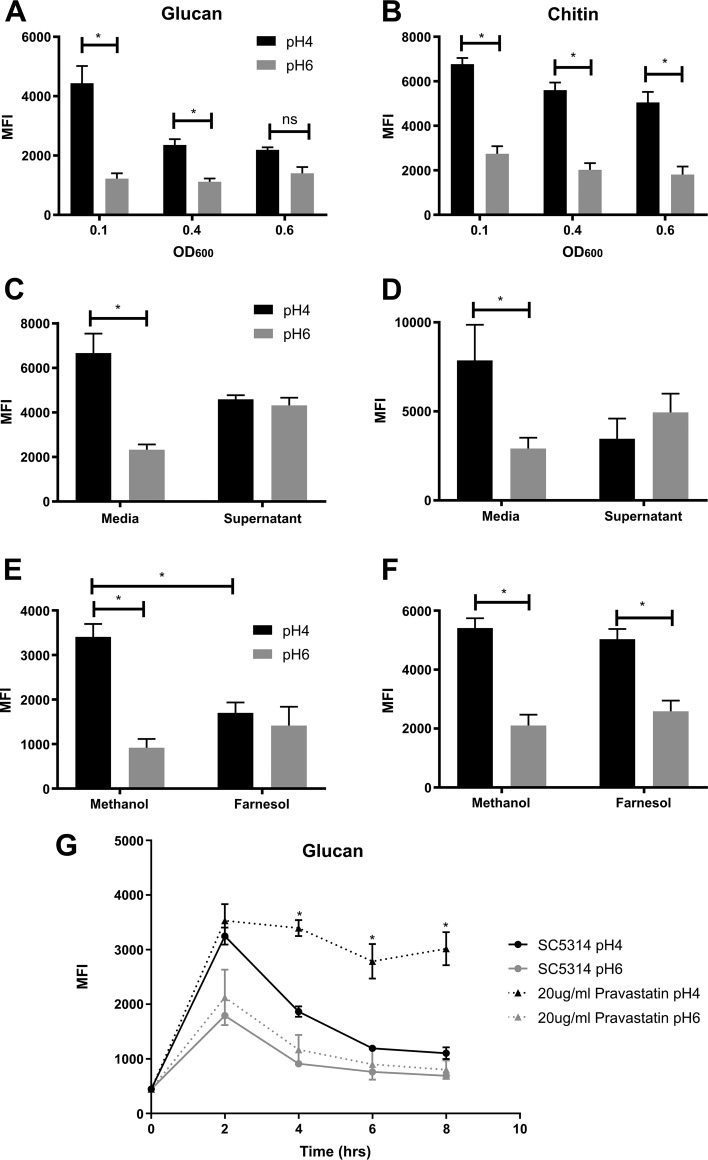
At high cell densities, the secretion of farnesol induces glucan remasking. Wild-type C. albicans (SC5314) was inoculated in fresh YPD buffered at pH 4 or pH 6 at an initial OD_600_ of 0.1, 0.4, or 0.6 and then incubated at 37°C for 4 h. Cells were then fixed and stained with FITC or TRITC label, and the median fluorescence intensities of exposed β1,3-glucan (A) and exposed chitin (B) were quantified by FACS. SC5314 was inoculated in sterile 8 h culture supernatant at an initial OD_600_ of 0.1 and glucan (C) and chitin (D) exposure quantified after 4 h of growth. SC5314 was inoculated in fresh YPD supplemented with 200 μM farnesol at an initial OD_600_ of 0.1 and glucan (E) and chitin (F) exposure quantified after 4 h of growth. (G) Addition of pravastatin to the medium prevented glucan remasking in YPD pH 4. Data represent the means and SEMs from three biological replicates and analyzed by 2-way ANOVAs with Sidak’s *post hoc* tests. *, *P* < 0.05.

As the cell density of the culture increases over time, we investigated whether the remasking of these PAMPs is cell density dependent. Inoculation of C. albicans at increasing cell densities had no effect on the exposure of chitin in response to low pH (Fig. 4B). However, increasing the starting inoculum of C. albicans had a modest effect on β-glucan exposure in response to acidic pH (Fig. 4A). To investigate whether this cell density-dependent regulation of PAMP exposure was mediated by a secreted molecule, culture supernatants from cells grown for 8 h (time point at which cell wall is restored) were filter sterilized, pH corrected, and reinoculated with C. albicans. Interestingly, supernatants from 8 h cultures significantly reduced both chitin and β-glucan exposure (Fig. 4C and D) at pH 4, but showed a modest increase at pH 6 compared to the control. However, wild-type C. albicans grew much slower in the 8 h supernatants compared to those in standard buffered YPD. Therefore, to decipher whether the effect on glucan exposure was due to nutrient restriction or the presence of a secreted signaling molecule, supernatants were supplemented with 2% glucose, 2% peptone, 1% yeast extract, and YPD to replace depleted nutrients. Supplementation of the supernatants improved the growth rate of C. albicans (Fig. S3), suggesting that the restricted growth was due to nutrient depletion. However, supplementation of the supernatants did not restore pH-dependent cell wall remodeling (Fig. S3), confirming that there is a role for secreted molecules in regulating cell wall remodeling.

10.1128/mBio.02347-19.3 FIG S3 Reduced cell wall remodelling in culture supernatants is not due to growth deficiencies. (A) Growth curves of SC5314 in fresh YPD medium, 1:1 ratio fresh medium and 8-h supernatant, or with supernatant replenished, buffered at pH 4 or pH 6 at 37°C. Wild-type strain (SC5314) was grown in fresh YPD (control), 1:1 ratio fresh medium and 8-h supernatant, or with supernatant supplemented with the indicated component, buffered at pH 4 or pH 6 at 37°C for 4 h. Cells were then fixed and stained for glucan (B) and chitin (C) exposure. Data represent the means and SEMs from three biological replicates and analyzed by 2-way ANOVA with Sidak’s *post hoc* tests. *, *P* < 0.05. Download FIG S3, TIF file, 0.4 MB. Copyright © 2019 Cottier et al. 2019 Cottier et al. This content is distributed under the terms of the Creative Commons Attribution 4.0 International license.

### Farnesol regulates remasking of glucan but not chitin.

At high cell densities, microbes secrete signaling molecules into the environment for communication purposes, which function to regulate virulence attributes. Farnesol is the only *bona fide* quorum sensing molecule known in C. albicans (36, 37). Therefore, we investigated whether cell density-dependent inhibition of cell wall remodeling is regulated via farnesol. Addition of exogenous farnesol did not affect pH-dependent chitin exposure (Fig. 4F), suggesting that a secreted factor other than farnesol regulates remasking of cell wall chitin. However, the addition of exogenous farnesol affected pH-dependent unmasking of β-glucan (Fig. 4E), reducing glucan exposure similar to that of the 8 h culture supernatants, suggesting that the active agent in the culture supernatant is the fungal quorum sensing molecule (QSM) farnesol. To confirm that β-glucan remasking is mediated via the secretion of farnesol, glucan exposure was quantified in the presence of pravastatin, a molecule known to impair farnesol production (38). Addition of pravastatin did not affect the growth rate of C. albicans (data not show). However, C. albicans cells grown in the presence of pravastatin did not undergo β-glucan remasking (Fig. 4G). Therefore, farnesol is one of the main effectors involved in the regulation of β-glucan exposure.

### Chitin is remasked by an unidentified, secreted, small heat-stable compound.

As farnesol had no impact of chitin remasking, we sought to identify the secreted molecule regulating chitin exposure. To determine whether the secreted factor was a protein or nucleotide, the supernatants were heat inactivated and treated with proteinase K, DNase, or RNase. However, supernatants devoid of secreted proteins and nucleotides still inhibited chitin exposure under acidic conditions, although pH 4 samples always had slightly more chitin exposure than pH 6 samples (Fig. S4A), suggesting that the secreted factor is a heat stable, nonproteinaceous secreted factor(s). To elucidate the hydrophobicity of the secreted molecule, hydrophobic molecules were removed from the supernatants using Amberlite. Supernatants containing only hydrophilic molecules still induced chitin remasking (Fig. S4A). To identify the size of the secreted molecule(s), the supernatants were size fractionated. Due to the size exclusion columns removing nutrients from the medium, filtered supernatants were diluted 1:1 with fresh buffered medium. Diluted supernatants maintained a modest effect on chitin exposure, which was maintained in fractions containing molecules smaller than 3 kDa, although pH 4 samples always had slightly more chitin exposure than pH 6 samples. On the other hand, supernatants containing only molecules larger than 3 kDa showed greater chitin exposure at pH 4 (Fig. S4B). Taken together, the data suggest that a secreted, small, heat-stable non-proteinaceous molecule(s) regulates chitin exposure in response to pH, although other factors may also play a role. Therefore, multiple processes regulate cell wall remasking, with chitin remasking induced by the secretion of an unidentified secreted, small, heat-stable, non-proteinaceous hydrophilic molecule and glucan exposure regulated by farnesol in a cell density-dependent manner.

10.1128/mBio.02347-19.4 FIG S4 Chitin remasking is mediated via a secreted small, heat-stable, nonproteinaceous hydrophilic molecule. Wild-type C. albicans (SC5314) was grown in fresh YPD (control), or in a 1:1 ratio of fresh medium and 8-h supernatant treated or not with proteinase K, DNase, RNase, or Amberlite or heat inactivated (A) and fractionated by a 3-kDa filter (B). Cells were then fixed and stained for chitin exposure. Data represent the means and SEMs from three biological replicates and analysed by 2-way ANOVA with Sidak’s *post hoc* tests. *, *P* < 0.05. Download FIG S4, TIF file, 0.5 MB. Copyright © 2019 Cottier et al. 2019 Cottier et al. This content is distributed under the terms of the Creative Commons Attribution 4.0 International license.

### A core set of genes is regulated by pH over time.

**FIG 5:**
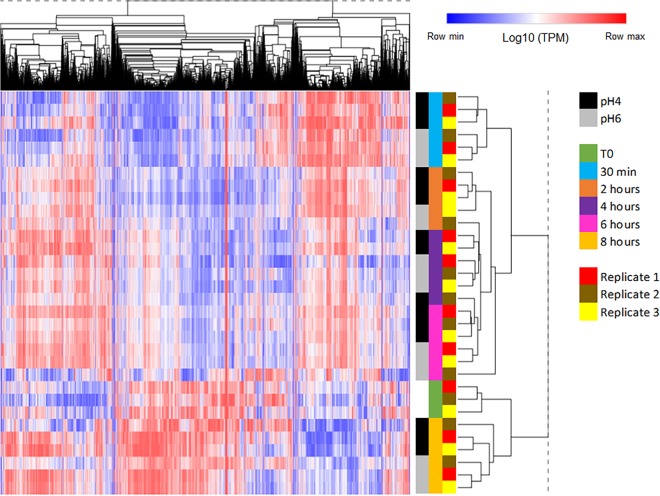
The global transcriptional profile of C. albicans is more influenced by time than environmental pH. Two-way hierarchical clustering of 6,218 transcripts (columns) and 32 samples (rows). Log_10_-transformed TPM expression values were converted to z-scores, with red indicating expression levels above and blue symbolizing expression levels below the mean expression level of each gene across the samples. Distance metric = 1 − Pearson correlation.

Masking of fungal cell wall glucan has been linked to changes in the global transcriptional profile of C. albicans in response to other environmental stimuli (29, 39). Therefore, to identify which genes are differentially regulated at pH 4, in both the unmasking and remasking phases, as well as to identify a putative pathway for the unknown molecule responsible for chitin remasking, a global transcriptional approach was taken. Total RNAs were extracted from samples at 0, 0.5, 2, 4, 6, and 8 h (*T*_0_, *T*_0.5_, *T*_2_, *T*_4_, *T*_6_, and *T*_8_, respectively) after inoculation in fresh media (pH 4 and pH 6) in triplicates. cDNA libraries were prepared for all 33 samples, and sequencing was performed using 2 × 50-bp reads. Quality controls were satisfactory for all samples except for sample pH 6_T2_1, which was then removed from subsequent analysis. Reads (average 6.27 × 10^7^ [± 1.35 × 10^7^] reads per sample) were mapped to the 6,214 transcripts present in assembly 21 of the C. albicans genome, and gene expression was reported as transcript per kilobase million (TPM) (see the supplemental material). Hierarchical clustering confirmed that all 32 samples clustered first by time of incubation, (i) early transcriptional response (0.5 h), (ii) intermediate response (2 to 6 h), and (iii) late response (*T*_0_ and 8 h), and then by pH (Fig. 5).

**FIG 6:**
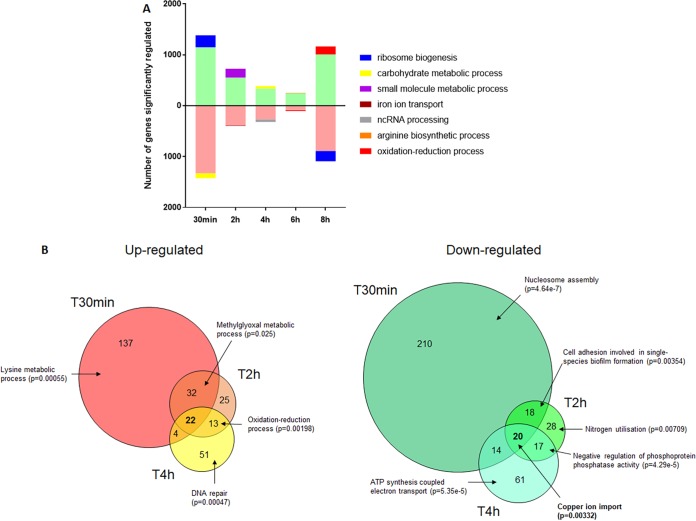
Identification of significantly regulated genes in response to time and environmental pH. (A) Genes significantly regulated between one time point to the next are represented, with main GO enrichment process reported for each up- and downregulated group. (B) Genes significantly regulated between pH 4 and pH 6 at *T*_0.5_ (T30min), *T*_2_ (T2h), and *T*_4_ (T4h). The main GO enrichment process of each group is reported when significant.

As the hierarchical clustering identified environmental pH as a minor modulator of the transcriptional responses, we performed a gene ontology (GO) enrichment analysis between time points by merging both pH sets together (Fig. 6A, see also Data set S1). By doing so, we followed the kinetics of gene regulation through time from one time point to the next. A large proportion of genes (45% of all genes) was significantly regulated between *T*_0_ and *T*_0.5_, with ribosome biogenesis being the most enriched GO group identified (*P* = 2.34 × 10^−98^) in upregulated genes, indicative of growth initiation. The number of genes differentially regulated decreased to 357 (5.7% of all genes) between *T*_4_ and *T*_6_. However, the number of differentially regulated genes increased to 2,255 (36% of the total) between *T*_6_ and *T*_8_ (Data set S1). GO enrichment confirmed that ribosome biogenesis was overrepresented (*P* = 1.66 × 10^−80^) in the downregulated genes (Fig. 6A), indicative of reduced growth. In contrast, aerobic respiration genes were significantly enriched in the set of upregulated genes (*P* = 6.04 × 10^−07^), indicative of the diauxic shift in C. albicans.

10.1128/mBio.02347-19.8 DATA SET S1 Transcript per kilobase million for each of the 6,214 transcripts under all 32 conditions, as well as lists of significantly regulated genes and GO enrichment for the reported one-to-one comparison. Download Data Set S1, XLSX file, 1.8 MB. Copyright © 2019 Cottier et al. 2019 Cottier et al. This content is distributed under the terms of the Creative Commons Attribution 4.0 International license.

To identify the transcriptional response of C. albicans associated with PAMP exposure in response to environmental pH, we compared the genes that were differentially regulated between pH 4 and pH 6 at each time point from *T*_0.5_ to *T*_8_. We found that the numbers of genes significantly regulated between both conditions were 485, 190, 223, 78, and 754, respectively (Data set S1). A GO enrichment analysis did not identify any term consistently enriched in all time points. We focused our attention on time points *T*_0.5_, *T*_2_, and *T*_4_, when PAMP unmasking occurred (Fig. 6B). We identified a core set of genes (42 genes in total) differentially regulated at *T*_0.5_, *T*_2_, and *T*_4_. From this core set of genes, 22 were significantly upregulated at pH 4 compared to at pH 6 (*IFD6*, *orf19.4476*, *HAK1*, *CSH1*, *orf19.3988*, *orf19.1430*, *RME1*, *CRZ2*, *orf19.7306*, *MDR1*, *orf19.851*, *orf19.5290*, *NAG3*, *orf19.2846*, *DAG7*, *orf19.4370*, *orf19.4690*, *HSP31*, *orf19.6586*, *HSX11*, *FET99*, and *BUL1*), while 20 genes were significantly downregulated (*orf19.1152*, *HGT6*, *orf19.7077*, *WOR3*, *orf19.6608*, *orf19.85*, *POL93*, *GAP4*, *PCK1*, *orf19.6079*, *orf19.751*, *orf19.4749*, *orf19.6077*, *SOD3*, *PHR1*, *CTR1*, *FET31*, *IHD1*, *FRE7*, and *FRE30*). GO term enrichment confirmed that this core set of pH-regulated genes was enriched for metal ion transport (*P* = 0.04661), with a particular emphasis on copper transport (Data set S1).

**FIG 7:**
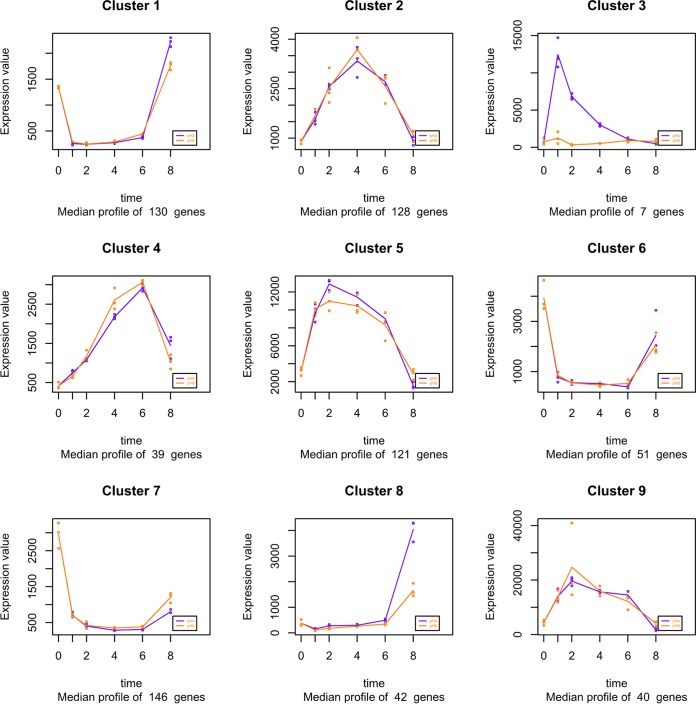
Cluster analysis identifies subsets of genes regulated through time and pH. *T*_0_ samples were used as a common starting point to the pH 4 (purple) and pH 6 (orange) series. Both pHs were evaluated simultaneously to determine the clusters using maSigPro.

To identify clusters of genes regulated by pH through time, a separate time course analysis was performed using maSigPro (40). This analysis identified 9 clusters of genes that shared similar expression profiles (Fig. 7). Most of the clusters identified showed regulation through time, but not pH, concurrent with our initial analysis, where time was identified as the main factor influencing gene expression. Interestingly, however, this analysis identified one cluster (cluster 3) in which gene expression was regulated by time and pH (Fig. 7). This cluster of genes was composed of 7 genes (*IFD6*, *orf19.1430*, *CRZ2*, *orf19.4476*, *DAG7*, *GDH3*, and *MNN42*), with all genes displaying enhanced expression at pH 4 at early time points (*T*_0.5_ to *T*_4_) compared to that at pH 6. However, at later time points, the expression of these 7 genes under acidic conditions declined to levels comparable to those of cells grown at pH 6. This gene expression profile mimics the identified pattern of PAMP exposure, indicating that these genes may be involved in regulating pH-dependent cell wall remodeling. Comparison of the two independent analyses identified a significant overlap of the genes identified as significantly differentially regulated by pH, and 6 of the 7 genes in cluster 3 (except *GDH3*) identified as part of the 42 core pH-regulated genes.

### Efg1 controls chitin exposure potentially via modulation of *CHT2* expression.

To investigate the role of these core pH-responsive genes in cell wall remodeling, nine mutants (*IFD6*, *CRZ2*, and *DAG7* identified by both analyses and *HAK1*, *FET99*, *HGT6*, *PHR1*, *FET31*, and *IHD1*, members of the core response) were selected for analysis of their cell wall structures. All tested strains maintained similar cell wall remodeling dynamics to the parental control strain, exhibiting initial β-glucan and chitin exposure, followed by rapid remasking of these PAMPs (Fig. S5), suggesting that the regulation of pH-dependent PAMP exposure is mediated by a complex mechanism. Therefore, we focused on identifying key transcription factors that are involved in the regulation of these 42 core pH-responsive genes. Using PathoYeastract (41), we identified seven transcription factors (*RIM101*, *SKN7*, *NDT80*, *EFG1*, *PHO4*, *CAS5*, and *TYE7*) that were strongly associated with the core pH response (*P* < 0.00005) and were predicted to regulate around half of the core genes. A screening of mutants defective in these key transcription factors did not identify strains with altered β-glucan exposure. However, inactivation of *EFG1* led to a significant reduction in chitin exposure at pH 4, at both 3 h and 7 h postinoculation (Fig. S5).

10.1128/mBio.02347-19.5 FIG S5 Individual genes regulated by environmental pH are not involved in pH-induced cell wall remodelling. C. albicans control (SN250) and mutant strains were inoculated in fresh YPD buffered at pH 4 or pH 6 at 37°C and grown for 3 and 7 h. Cells were then fixed and stained for glucan (A, B) and chitin (C, D) exposure. Data represent the means and SEMs from three biological replicates and analysed by 2-way ANOVA with Sidak’s *post hoc* tests. *, *P* < 0.05. Download FIG S5, TIF file, 2.0 MB. Copyright © 2019 Cottier et al. 2019 Cottier et al. This content is distributed under the terms of the Creative Commons Attribution 4.0 International license.

**FIG 8:**
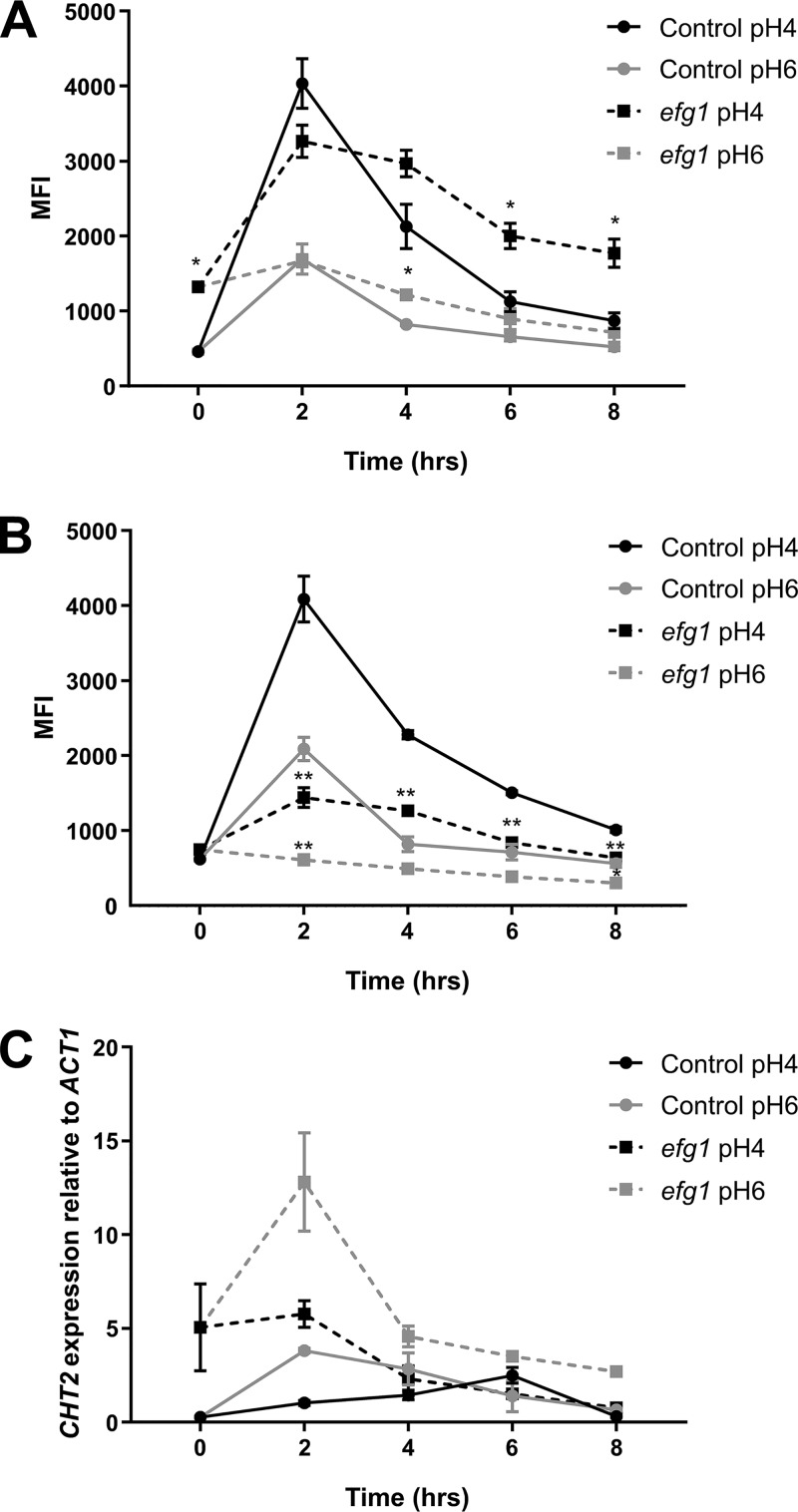
Efg1 regulates chitin exposure through the regulation of *CHT2*. C. albicans control (SN250) and *EFG1* mutant strains were inoculated in fresh YPD buffered at pH 4 or pH 6 at 37°C. Cells were then fixed and stained for exposed β1,3-glucan (A) and chitin (B). (C) Cells from the same culture were used to extract RNA and measure *CHT2* expression by semiquantitative reverse transcription PCR (RT-PCR), with expression levels normalized to those of *ACT1*. Data represent the means and SEMs from three biological replicates and analyzed by 2-way ANOVAs with Sidak’s *post hoc* tests.*, *P* < 0.05.

To elucidate the role of Efg1 in regulating pH-dependent cell wall remodeling, we measured glucan and chitin exposure over an 8 h period in the *efg1* null strain at pH 4 and pH 6. Deletion of *EFG1* did not affect the timing of β-glucan exposure (Fig. 8A). In contrast, however, chitin exposure was significantly reduced in the *efg1* mutant throughout the times tested and at all pHs (Fig. 8B). Therefore, Efg1 plays a critical role regulating genes involved in chitin exposure.

We previously demonstrated that the fungal chitinase Cht2 is involved in C. albicans chitin exposure, with reduced *CHT2* expression promoting chitin exposure (28). Therefore, we analyzed the expression of *CHT2* in response to environmental pH through time in both wild-type and *efg1* mutant strains. In agreement with our previous data, the expression of *CHT2* in wild-type cells was low initially and gradually increased over time. However, in the *efg1* mutant, *CHT2* was significantly derepressed (Fig. 8C), confirming that Efg1-dependent *CHT2* expression is involved in chitin concealing within the cell wall.

## DISCUSSION

Immune evasion is a powerful mechanism for commensal organisms to avoid triggering an immune response when colonizing the host (30, 31). As C. albicans has coevolved with the human host for millennia, the development of strategies to evade the innate immune system is necessary for the fungus to maintain its commensal state within the host. The most recently identified strategy C. albicans used to manipulate the host’s innate immune response is through remodeling its outer surface, the fungal cell wall, to hide or reveal specific PAMPs. This process has been coined cell wall masking (the concealment of PAMPs) and cell wall unmasking (exposure of PAMPs), with the most important PAMP being β-glucan. Indeed, β-glucan is recognized by dectin-1, and this interaction plays a major role in antifungal immunity (19, 33). Therefore, any masking or unmasking of this molecule significantly affects the host’s innate immune response (42).

Here, we show that cell wall remodeling in response to pH is a transient process requiring periods of unmasking and masking. Although other environmental conditions are known to induce cell wall remodeling, the dynamics of these responses have not been investigated in detail, with the majority of observations made at a single time point. Longitudinal studies with lactate suggest that in this scenario, β-glucan masking is constant (29), but the impact of growth rate and subsequent passaging of cells was not investigated. Given that during an infection, C. albicans will be exposed to multiple environmental signals both sporadically and in parallel, understanding how these signals are integrated to shape the fungal cell surface presented to the innate immune system is paramount. For example, during colonization of the vaginal mucosa, C. albicans will be exposed to an environment of low pH, with lactate as the main carbon source, and will experience periods of hypoxia. Lactate and hypoxia induce masking of glucan (29–31), while low pH promotes glucan exposure. Staining of the fungal cell wall from vaginal swabs suggests that, *in vivo*, the glucan is mainly concealed from the innate immune system (32), favoring the effects mediated by lactate and hypoxia. However, for these swabs, the period of colonization and *in vivo* growth rate are unknown. Given that PAMP exposure fluctuates over time, it is hard to correlate PAMP exposure with virulence from a single swab. In our G. mellonella 6-day infection model, C. albicans cells adapted to acidic environments were more pathogenic than cells adapted to pH 6, despite cells grown at pH 4 exhibiting higher phagocytosis rates. Given that acid-adapted cells survive better inside the neutrophil phagosome and are associated with a stronger proinflammatory innate immune response, we propose that during infection, initial pH-dependent PAMP exposure will cause C. albicans to elicit a strong proinflammatory innate immune response, recruiting neutrophils and macrophages to the site of infection, which phagocytose but do not kill the pathogen. This strong proinflammatory environment, at least in the G. mellonella infection model, appears to be maintained and results in increased host damage, enhancing fungal pathogenicity. Therefore, in our model system at least, it is the initial immune response to PAMP exposure that determines the outcome of the infection, and the concealing of PAMPs at later time points cannot compensate for the initiation of the innate immune response and incurred host damage.

In C. albicans, the remasking of the fungal cell wall is mediated via the secretion of farnesol and an unidentified, small, heat-stable, nonproteinaceous secreted molecule. In agreement with farnesol playing a role in cell wall remodeling, several genes involved in farnesol biosynthesis (i.e., *DPP1* and *ERG20*) were upregulated at the later time points, when the cell wall was being remasked. Farnesol is known to increase mitochondrial ROS production (43), increasing the resistance of C. albicans to subsequent ROS stress. Under hypoxic conditions, elevated ROS levels result in increased masking of glucan through modulation of the cAMP-PKA pathway (30). Therefore, at high cell densities, farnesol may function to increase intercellular ROS and promote glucan remasking through cAMP-PKA signaling. Although intracellular ROS levels initially correlated with pH-induced glucan exposure, this correlation did not hold up at the later time points when glucan was remasked. In agreement with this, disruption of *TPK1* or *TPK2* did not affect pH-induced glucan exposure. Disruption of *CYR1* and *TPK1-TPK2* together did perturb glucan exposure; however, the fitness of the cells was also dramatically affected, making it hard to discern whether this observation was truly an effect of the altered cell signaling or a consequence of reduced fitness. So far, ROS signaling has not been linked to β-glucan masking in response to lactate, suggesting that cell wall remodeling is a complex phenotype regulated by multiple processes.

Our global transcriptional analysis revealed that temporal regulation was predominant over environmental pH. As expected from previous studies (44), inoculation in fresh medium of overnight grown cells resulted in a significant upregulation of genes involved in ribosome biogenesis, which declined at later time points. Unfortunately, we could not identify any GO enrichment linked to the remasking mechanism of both PAMPs over time. However, we discovered a core group of 42 genes that were differentially regulated in response to pH during a period of time when significant differences in PAMP exposure were observed. Interestingly, 3 of these genes (*HAK1*, *CRZ2*, and *FET99*) are downregulated during lactate-induced glucan masking (29) but induced in response to acidic pH, where β-glucan becomes exposed. However, deletion of these genes individually did not perturb pH-induced cell wall remodeling (see Fig. S5 in the supplemental material). It remains to be elucidated whether inactivation of multiple pH-responsive genes perturbs pH-dependent PAMP exposure. Nonetheless, we found that the transcriptional factor Efg1 directly or indirectly regulates 25 of the 42 genes and that inactivation of *EFG1* induced a significant reduction in chitin unmasking in acidic environments. This observation is partially attributed to increased *CHT2* expression (45), a key cell wall remodeling enzyme known to control chitin exposure in C. albicans (28).

Each *Candida* species varied the amount of glucan exposed on its surface, with C. auris and C. tropicalis displaying the highest exposure of glucan. This increase in glucan exposure correlates with phagocytosis rates, with *Candida* cells with more glucan exposure at the surface phagocytosed at higher rates (29, 30, 33). C. auris also displayed significantly more chitin at the cell wall periphery than the other *Candida* species. Considering the predominate roles glucan and chitin play in mediating the host-pathogen interaction on host immune response (18), these cell wall modifications could play key roles in the virulence of C. auris. Interestingly, pH-dependent glucan unmasking was only observed in dimorphic *Candida* species, with yeast-locked non-*albicans Candida* strains not displaying cell wall remodeling under our tested conditions. However, it remains to be investigated whether the molecular mechanisms involved in hyphal formation share a function in cell wall remodeling. During colonization of the human host, C. albicans faces various environmental changes to which it must adapt in order to either maintain its position within the microbiota or to cause infection. Many of these environmental cues are strong promoters of C. albicans morphogenesis but also function to regulate the structure and composition of the fungal cell wall. Given that cell wall remodeling is a dynamic response, which is regulated through the secretion of signaling molecules, understanding how C. albicans adapts to the host overtime and how all these signals are integrated to modulate the host-pathogen interaction will provide new insight in the development of effective compounds against this opportunistic pathogen.

## MATERIALS AND METHODS

### Strains and culture conditions.

Strains used in this study are summarized in Table S1 in the supplemental material. Yeast strains were grown in YPD medium (50 g/liter YPD broth; Sigma-Aldrich) supplemented with 3.57% HEPES and buffered to pH 4 or pH 6 with HCl. Cultures were incubated at 37°C, 200 rpm, unless stated otherwise.

10.1128/mBio.02347-19.6 TABLE S1 Strains used in this study. Download Table S1, DOCX file, 0.1 MB. Copyright © 2019 Cottier et al. 2019 Cottier et al. This content is distributed under the terms of the Creative Commons Attribution 4.0 International license.

### Immunofluorescence staining of cell wall components.

To quantify the exposure of fungal cell wall PAMPs, C. albicans cells were stained with specific cell wall probes and quantified by immunofluorescence. In brief, yeast strains were grown overnight in YPD at 37°C, or 30°C for S. cerevisiae, at 200 rpm. Cells were subcultured in fresh medium at a starting optical density at 600 nm (OD_600_) of 0.1, grown from 30 min to 8 h at 37°C and 200 rpm, harvested by centrifugation at 4,000 rpm for 3 min, fixed on ice for 30 min in 4% paraformaldehyde (PFA) in phosphate-buffered saline (PBS), and washed three times in PBS. To stain for surface-exposed β-1,3-glucan, PFA-fixed yeast cells were incubated with 3 μg/ml Fc-dectin-1 (a kind gift from G. Brown, University of Aberdeen) and goat anti-human IgG Fc conjugated to Alexa Fluor 488 (Invitrogen). Surface-exposed chitin was stained concurrently by the addition of 50 μg/ml tetramethyl rhodamine isocyanate (TRITC)-conjugated wheat germ agglutinin (WGA) (Molecular Probes, Life Technologies). Mannan was stained using 50 μg/ml TRITC-conjugated concanavalin A (Thermo Fisher Scientific). Cells were analyzed on an Attune fluorescence-activated cell sorter (FACS) machine (50 mW blue/violet standard configuration), with 10,000 events observed. Fluorescein isothiocyanate (FITC)-labeled cells were quantified using the 488-nm laser in combination with 530/30 and 555DLP filters, and TRITC fluorescence was quantified using the 488-nm laser in combination with 574/26 and 650DLP filters. The median fluorescence intensity (MFI) was corrected for fluorescence compensation and background fluorescence.

### Supernatant experiments.

To access the role of secreted molecules in cell wall remodeling, C. albicans was inoculated in YPD buffered to either pH 4 or pH 6 to an OD_600_ of 0.1 and grown at 37°C, 200 rpm, for 8 h. Supernatants were cleared by centrifugation and supplemented with medium components where specified. The pH was checked and readjusted with HCl if required, and then the supernatants were filter sterilized. To heat inactivate the supernatant, the supernatants were incubated at 70°C for 1 h. To remove any secreted nucleotides or proteins, the supernatants were treated with 50 μg/ml DNase 1, 50 μg/ml RNase A, or 50 μg/ml proteinase K for 1 h. Supernatants were then inoculated with fresh C. albicans to an OD_600_ of 0.1, and cultures were incubated for 4 h. To size fractionate the supernatants, 10 ml of supernatant was loaded into a size exclusion spin column with a 3-kDa cutoff, and samples were centrifuged according to the manufacturer’s recommendations. The flowthrough containing molecules smaller than 3 kDa was diluted 1:1 with fresh buffered YPD. Larger molecules were resuspended in complete buffered YPD. Supernatants were then inoculated with fresh C. albicans to an OD_600_ of 0.1, and cultures were incubated for 4 h.

### Farnesol and pravastatin treatment.

Mixed-isomer farnesol (Sigma) was diluted 1:10 in 100% methanol and then added to cultures at a final concentration of 200 μM. Control cultures were supplemented with an equal volume of 100% methanol. Pravastatin was diluted in water and used in assays at a final concentration of 200 μM.

### pHluorin microtiter plate assay.

Intracellular pH was measured using the pH-responsive green fluorescent protein (GFP) variant pHluorin as previously described (46). In brief, cells expressing the empty vector or expressing cytoplasmic PHL2 were grown overnight in YPD at 37°C. Cells suspensions were diluted 1/1,000 in fresh buffered minimal medium (yeast nitrogen base [YNB]) prepared at pH 4 or pH 6. Aliquots of 200 μl were added to 96-well plates in duplicates for each strain in each medium. Using a Cytation 5 plate reader, continuous measurements were acquired for growth (OD_600_) and pHluorin detection every 20 min. Relative fluorescence units (RFU) were obtained with an emission filter at 509 nm (9-nm bandwidth) and excitation wavelengths of 395 nm and 470 nm (both with 9-nm bandwidth). Cells expressing the empty vector were used to measure background fluorescence, which was subtracted from the RFU obtained from cells expressing the PHL2-based indicators. The I_395_/I_470_ ratios were calculated as an average and standard deviation from two replicates.

### RNA preparation and sequencing.

The global transcriptional response of C. albicans to acidic environments was evaluated by transcriptome sequencing (RNA-Seq). Overnight cultures were diluted to an OD_600_ of 0.1 and grown at 37°C, 200 rpm, for 30 min, 2 h, 4 h, 6 h, or 8 h in YPD buffered at pH 4 or pH 6. Cells were harvested by centrifugation, and equivalent amounts of starting material were flash-frozen in liquid nitrogen. Total RNA was extracted using an RNeasy kit (Qiagen) according to the manufacturer’s recommendations. Extracted RNA quantity was assessed on a NanoDrop 8000 spectrophotometer (ND-8000-GL; Thermo Fisher). RNA quality and integrity were assessed using the Agilent TapeStation 2200 (Agilent G2964AA) with High Sensitivity RNA ScreenTapes (Agilent 5067-5579). Samples with an RNA integrity number greater than 8.0 were retained for downstream analysis. Total RNA (500 ng) was poly(A) selected prior to library construction using the NEBNext Poly(A) mRNA Magnetic Isolation Module (E7490L; New England BioLabs). RNA libraries were prepared using a NEBNext Ultra Directional RNA Library Prep kit (E7420L; New England BioLabs) and NEBnext Multiplex Oligos Illumina-compatible Dual Index primers (E7600S; New England BiolLabs), with the support of a Biomek FxP liquid handling robot (A31842; Beckman Coulter) at the Environmental Omics Sequencing Facility (University of Birmingham, UK). The quality of the RNA libraries was assessed on a TapeStation 2200 (Agilent G2964AA) with High Sensitivity D1000 DNA ScreenTape (Agilent 5067-5584). Equimolar multiplexed libraries (paired end [PE], 50 bp) were sequenced on an Illumina HiSeq 4000 platform.

### RNA-Seq data analysis.

For sample group comparisons, reads were analyzed using the RNA-sequencing package from CLC Genomic workbench 11.0.1 (Qiagen). Reads were processed for adapter and quality trimming before being mapped to C. albicans reference genome (assembly 21, version s02-m09-r10). The expression level of each of the 6,214 open reading frames (ORFs) was reported as transcript per kilobase million (TPM). Statistical analysis was performed after the addition of the lowest TPM measurement to all values. The data were log_10_ transformed, and differential expression between conditions was considered significant if the absolute value fold change was >2 and false-discovery rate (FDR) was <0.01. Hierarchical clustering was performed on Morpheus (https://software.broadinstitute.org/morpheus/). CGD GO term finder (47) was used to perform gene ontology (GO) analysis with *P* values corresponding to Bonferroni-corrected hypergeometric test *P* values.

For the time course analysis, prior to mapping, Illumina adaptor sequences were removed from the reads using Trimmomatic (v0.36-5). Reads were then aligned to the C. albicans build 21 reference genome using STAR (v2.5.2b). Read counts were loaded into R (v3.3.2) for differential gene expression analysis. Significant numbers of reads were detected for 6,161 annotated ORFs. Time course analysis was carried out using maSigPro (v1.56.0) with default parameters taking *T*_0_ as a common starting point for both pH 4 and pH 6 (40).

### Quantitative reverse transcription PCR.

Samples were prepared according to the manufacturer’s recommendations (qPCR SyGreen 1-Step Detect Lo-ROX; PCR Biosystems). Each reaction was performed with 50 ng of RNA, using primers described in Table S2, in a Bio-Rad CFX Connect real-time PCR detection system.

10.1128/mBio.02347-19.7 TABLE S2 Primers used in this study. Download Table S2, DOCX file, 0.1 MB. Copyright © 2019 Cottier et al. 2019 Cottier et al. This content is distributed under the terms of the Creative Commons Attribution 4.0 International license.

### Superoxide detection assay.

To measure the intracellular level of superoxide, cells were grown in the designated media. Then, equivalent volumes at an OD_600_ of 0.3 were centrifuged 3 min at 4,000 rpm, and the pellets were washed once in PBS. Cells were then mixed with superoxide detection reagent (ROS-ID Total ROS/Superoxide detection kit; Enzo) and mounted for microscopy. Fluorescence was quantified using Zen 2 software.

### Phagocytosis assay.

J774.1A macrophages (Sigma-Aldrich, UK) were maintained in Dulbecco’s modified Eagle medium (DMEM) supplemented with 10% fetal bovine serum (FBS), 100 mM l-glutamine, and 100 mM penicillin/streptomycin at 37°C, 5% CO_2_. Then, 1 × 10^5^ J774.1A macrophages were seeded onto 13-mm-diameter glass coverslips in 24-well plates and allowed to attach for 24 h. Immediately before phagocytosis assays were performed, J774.1A macrophages were serum starved in serum-free DMEM for 1 h with 1.5 μg/ml phorbol myristate acetate (PMA). Yeast cells were inoculated in YPD and grown overnight at 37°C, 200 rpm. Cells were subcultured in YPD buffered at the appropriate pH to an OD_600_ of 0.1 and grown for the reported time at 37°C, 200 rpm. Cells were harvested, washed three times in sterile endotoxin-free PBS (Sigma-Aldrich, UK), and resuspended in PBS to 1 × 10^7^ cells/ml. PMA-containing medium was aspirated from the macrophages and replaced with fresh serum-free DMEM, to which 5 × 10^5^ *Candida* cells were added (multiplicity of infection [MOI] of 5). Cells were coincubated for 1 h, nonphagocytosed *Candida* cells were removed by repeated washing with sterile PBS, and cells were fixed with 4% PFA for 15 min. To distinguish between attached and phagocytosed yeasts, coverslips were stained for 30 min with 50 μg/ml ConA conjugated to FITC (Molecular Probes, Life Technologies), washed three times with PBS, and imaged using a Nikon TE2000. At least six images were taken per sample, with approximately 100 macrophages/image. Phagocytosis was scored in ImageJ. *Candida* cells stained with ConA-FITC were considered attached to the exterior of the macrophage, while nonstained yeasts were considered internalized and phagocytosed.

### Galleria mellonella infection model.

Experiments were performed as described previously (48). In brief, C. albicans was grown at 37°C in YPD buffered at the required pH and then washed twice in PBS. Yeast concentration was calculated using a hemocytometer and adjusted to a final concentration of 2 × 10^5^ cells/μl. G. mellonella larvae were then inoculated with 20 μl of PBS or yeast solution in their left penultimate prolegs and incubated at 30°C for 6 days. Survival was monitored daily.

### Neutrophil killing assay.

Neutrophils were isolated from peripheral blood as described previously (28). Neutrophils were coincubated with C. albicans at an MOI of 0.5 for 3 h, lysed with water, and plated to determine viable CFU counts compared to the viability of samples incubated in the absence of phagocytes.

### Statistical analysis.

Unless stated otherwise, data represent the mean ± standard errors of the mean (SEMs) from at least 3 biological replicates. Significant differences were calculated using 2-way analyses of variance (ANOVA) with Sidak’s *post hoc* tests on GraphPad Prism 8.

### Data availability.

Sequencing reads are available at the Gene Expression Omnibus (GEO) database (https://www.ncbi.nlm.nih.gov/geo/) at the following accession number: GSE130948.

## References

[1] Fedele G, Schiavoni I, Adkins I, Klimova N, Sebo P 2017 Invasion of dendritic cells, macrophages and neutrophils by the *Bordetella* adenylate cyclase toxin: a subversive move to fool host immunity. Toxins (Basel) 9:293. doi:10.3390/toxins9100293. PMC5666340 28934122

[2] Reyes A, Semenkovich NP, Whiteson K, Rohwer F, Gordon JI 2012 Going viral: next-generation sequencing applied to phage populations in the human gut. Nat Rev Microbiol 10:607–617. doi:10.1038/nrmicro2853. 22864264 PMC3596094

[3] Cianci R, Pagliari D, Piccirillo CA, Fritz JH, Gambassi G 2018 The microbiota and immune system crosstalk in health and disease. Mediators Inflamm 2018:2912539. doi:10.1155/2018/2912539. 29849485 PMC5937375

[4] Belkaid Y, Hand TW 2014 Role of the microbiota in immunity and inflammation. Cell 157:121–141. doi:10.1016/j.cell.2014.03.011. 24679531 PMC4056765

[5] Ehrt S, Schnappinger D 2009 Mycobacterial survival strategies in the phagosome: defence against host stresses. Cell Microbiol 11:1170–1178. doi:10.1111/j.1462-5822.2009.01335.x. 19438516 PMC3170014

[6] Charlier C, Nielsen K, Daou S, Brigitte M, Chretien F, Dromer F 2009 Evidence of a role for monocytes in dissemination and brain invasion by *Cryptococcus neoformans*. Infect Immun 77:120–127. doi:10.1128/IAI.01065-08. 18936186 PMC2612285

[7] Ward TL, Dominguez-Bello MG, Heisel T, Al-Ghalith G, Knights D, Gale CA 2018 Development of the human mycobiome over the first month of life and across body sites. mSystems 3:e00140-17. doi:10.1128/mSystems.00140-17. 29546248 PMC5840654

[8] Cottier F, Srinivasan KG, Yurieva M, Liao W, Poidinger M, Zolezzi F, Pavelka N 2018 Advantages of meta-total RNA sequencing (MeTRS) over shotgun metagenomics and amplicon-based sequencing in the profiling of complex microbial communities. NPJ Biofilms Microbiomes 4:2. doi:10.1038/s41522-017-0046-x. 29367879 PMC5773663

[9] Huffnagle GB, Noverr MC 2013 The emerging world of the fungal microbiome. Trends Microbiol 21:334–341. doi:10.1016/j.tim.2013.04.002. 23685069 PMC3708484

[10] Goncalves B, Ferreira C, Alves CT, Henriques M, Azeredo J, Silva S 2016 Vulvovaginal candidiasis: epidemiology, microbiology and risk factors. Crit Rev Microbiol 42:905–927. doi:10.3109/1040841X.2015.1091805. 26690853

[11] Yapar N 2014 Epidemiology and risk factors for invasive candidiasis. Ther Clin Risk Manag 10:95–105. doi:10.2147/TCRM.S40160. 24611015 PMC3928396

[12] Calderone RA, Fonzi WA 2001 Virulence factors of *Candida albicans*. Trends Microbiol 9:327–335. doi:10.1016/S0966-842X(01)02094-7. 11435107

[13] Cottier F, Muhlschlegel FA 2009 Sensing the environment: response of *Candida albicans* to the X factor. FEMS Microbiol Lett 295:1–9. doi:10.1111/j.1574-6968.2009.01564.x. 19473245

[14] Moyes DL, Wilson D, Richardson JP, Mogavero S, Tang SX, Wernecke J, Hofs S, Gratacap RL, Robbins J, Runglall M, Murciano C, Blagojevic M, Thavaraj S, Forster TM, Hebecker B, Kasper L, Vizcay G, Iancu SI, Kichik N, Hader A, Kurzai O, Luo T, Kruger T, Kniemeyer O, Cota E, Bader O, Wheeler RT, Gutsmann T, Hube B, Naglik JR 2016 Candidalysin is a fungal peptide toxin critical for mucosal infection. Nature 532:64–68. doi:10.1038/nature17625. 27027296 PMC4851236

[15] Rizzetto L, Weil T, Cavalieri D 2015 Systems level dissection of *Candida* recognition by dectins: a matter of fungal morphology and site of infection. Pathogens 4:639–661. doi:10.3390/pathogens4030639. 26308062 PMC4584279

[16] Casanova M, Lopez-Ribot JL, Martinez JP, Sentandreu R 1992 Characterization of cell wall proteins from yeast and mycelial cells of *Candida albicans* by labelling with biotin: comparison with other techniques. Infect Immun 60:4898–4906. 1383159 10.1128/iai.60.11.4898-4906.1992 PMC258246

[17] Mormeneo S, Marcilla A, Iranzo M, Sentandreu R 1994 Structural mannoproteins released by beta-elimination from *Candida albicans* cell walls. FEMS Microbiol Lett 123:131–136. doi:10.1111/j.1574-6968.1994.tb07212.x. 7988880

[18] Hall RA 2015 Dressed to impress: impact of environmental adaptation on the *Candida albicans* cell wall. Mol Microbiol 97:7–17. doi:10.1111/mmi.13020. 25846717 PMC4973840

[19] Brown GD, Herre J, Williams DL, Willment JA, Marshall AS, Gordon S 2003 Dectin-1 mediates the biological effects of beta-glucans. J Exp Med 197:1119–1124. doi:10.1084/jem.20021890. 12719478 PMC2193964

[20] Netea MG, Van Der Graaf CA, Vonk AG, Verschueren I, Van Der Meer JW, Kullberg BJ 2002 The role of toll-like receptor (TLR) 2 and TLR4 in the host defense against disseminated candidiasis. J Infect Dis 185:1483–1489. doi:10.1086/340511. 11992285

[21] van de Veerdonk FL, Marijnissen RJ, Kullberg BJ, Koenen HJ, Cheng SC, Joosten I, van den Berg WB, Williams DL, van der Meer JW, Joosten LA, Netea MG 2009 The macrophage mannose receptor induces IL-17 in response to *Candida albicans*. Cell Host Microbe 5:329–340. doi:10.1016/j.chom.2009.02.006. 19380112

[22] McGreal EP, Rosas M, Brown GD, Zamze S, Wong SY, Gordon S, Martinez-Pomares L, Taylor PR 2006 The carbohydrate-recognition domain of dectin-2 is a C-type lectin with specificity for high mannose. Glycobiology 16:422–430. doi:10.1093/glycob/cwj077. 16423983

[23] Wells CA, Salvage-Jones JA, Li X, Hitchens K, Butcher S, Murray RZ, Beckhouse AG, Lo YL, Manzanero S, Cobbold C, Schroder K, Ma B, Orr S, Stewart L, Lebus D, Sobieszczuk P, Hume DA, Stow J, Blanchard H, Ashman RB 2008 The macrophage-inducible C-type lectin, mincle, is an essential component of the innate immune response to *Candida albicans*. J Immunol 180:7404–7413. doi:10.4049/jimmunol.180.11.7404. 18490740

[24] Smeekens SP, van de Veerdonk FL, Kullberg BJ, Netea MG 2013 Genetic susceptibility to *Candida* infections. EMBO Mol Med 5:805–813. doi:10.1002/emmm.201201678. 23629947 PMC3779444

[25] Netea MG, Brown GD, Kullberg BJ, Gow NA 2008 An integrated model of the recognition of *Candida albicans* by the innate immune system. Nat Rev Microbiol 6:67–78. doi:10.1038/nrmicro1815. 18079743

[26] Gantner BN, Simmons RM, Underhill DM 2005 Dectin-1 mediates macrophage recognition of *Candida albicans* yeast but not filaments. EMBO J 24:1277–1286. doi:10.1038/sj.emboj.7600594. 15729357 PMC556398

[27] Hopke A, Nicke N, Hidu EE, Degani G, Popolo L, Wheeler RT 2016 Neutrophil attack triggers extracellular trap-dependent candida cell wall remodeling and altered immune recognition. PLoS Pathog 12:e1005644. doi:10.1371/journal.ppat.1005644. 27223610 PMC4880299

[28] Sherrington SL, Sorsby E, Mahtey N, Kumwenda P, Lenardon MD, Brown I, Ballou ER, MacCallum DM, Hall RA 2017 Adaptation of *Candida albicans* to environmental pH induces cell wall remodelling and enhances innate immune recognition. PLoS Pathog 13:e1006403. doi:10.1371/journal.ppat.1006403. 28542528 PMC5456412

[29] Ballou ER, Avelar GM, Childers DS, Mackie J, Bain JM, Wagener J, Kastora SL, Panea MD, Hardison SE, Walker LA, Erwig LP, Munro CA, Gow NA, Brown GD, MacCallum DM, Brown AJ 2016 Lactate signalling regulates fungal beta-glucan masking and immune evasion. Nat Microbiol 2:16238. doi:10.1038/nmicrobiol.2016.238. 27941860 PMC5704895

[30] Pradhan A, Avelar GM, Bain JM, Childers DS, Larcombe DE, Netea MG, Shekhova E, Munro CA, Brown GD, Erwig LP, Gow NAR, Brown A 2018 Hypoxia promotes immune evasion by triggering beta-glucan masking on the candida albicans cell surface via mitochondrial and camp-protein kinase A signaling. mBio 9:e01318-18. doi:10.1128/mBio.01318-18. 30401773 PMC6222127

[31] Lopes JP, Stylianou M, Backman E, Holmberg S, Jass J, Claesson R, Urban CF 2018 Evasion of immune surveillance in low oxygen environments enhances *Candida albicans* virulence. mBio 9:e02120-18. doi:10.1128/mBio.02120-18. PMC6222133 30401781

[32] Pericolini E, Perito S, Castagnoli A, Gabrielli E, Mencacci A, Blasi E, Vecchiarelli A, Wheeler RT 2018 Epitope unmasking in vulvovaginal candidiasis is associated with hyphal growth and neutrophilic infiltration. PLoS One 13:e0201436. doi:10.1371/journal.pone.0201436. 30063729 PMC6067721

[33] Gow NA, Netea MG, Munro CA, Ferwerda G, Bates S, Mora-Montes HM, Walker L, Jansen T, Jacobs L, Tsoni V, Brown GD, Odds FC, Van der Meer JW, Brown AJ, Kullberg BJ 2007 Immune recognition of *Candida albicans* beta-glucan by dectin-1. J Infect Dis 196:1565–1571. doi:10.1086/523110. 18008237 PMC2655640

[34] Hollomon JM, Grahl N, Willger SD, Koeppen K, Hogan DA 2016 Global role of cyclic AMP signaling in pH-dependent responses in *Candida albicans*. mSphere 1:e00283-16. doi:10.1128/mSphere.00283-16. 27921082 PMC5137381

[35] Brown AJ, Brown GD, Netea MG, Gow NA 2014 Metabolism impacts upon *Candida* immunogenicity and pathogenicity at multiple levels. Trends Microbiol 22:614–622. doi:10.1016/j.tim.2014.07.001. 25088819 PMC4222764

[36] Polke M, Leonhardt I, Kurzai O, Jacobsen ID 2018 Farnesol signalling in *Candida albicans* - more than just communication. Crit Rev Microbiol 44:230–243. doi:10.1080/1040841X.2017.1337711. 28609183

[37] Hornby JM, Jensen EC, Lisec AD, Tasto JJ, Jahnke B, Shoemaker R, Dussault P, Nickerson KW 2001 Quorum sensing in the dimorphic fungus *Candida albicans* is mediated by farnesol. Appl Environ Microbiol 67:2982–2992. doi:10.1128/AEM.67.7.2982-2992.2001. 11425711 PMC92970

[38] Tashiro M, Kimura S, Tateda K, Saga T, Ohno A, Ishii Y, Izumikawa K, Tashiro T, Kohno S, Yamaguchi K 2012 Pravastatin inhibits farnesol production in *Candida albicans* and improves survival in a mouse model of systemic candidiasis. Med Mycol 50:353–360. doi:10.3109/13693786.2011.610037. 21954955

[39] Duvenage L, Walker LA, Bojarczuk A, Johnston SA, MacCallum DM, Munro CA, Gourlay CW 2019 Inhibition of classical and alternative modes of respiration in *Candida albicans* leads to cell wall remodeling and increased macrophage recognition. mBio 10:e02535-18. doi:10.1128/mBio.02535-18. 30696734 PMC6355986

[40] Conesa A, Nueda MJ, Ferrer A, Talon M 2006 maSigPro: a method to identify significantly differential expression profiles in time-course microarray experiments. Bioinformatics 22:1096–1102. doi:10.1093/bioinformatics/btl056. 16481333

[41] Monteiro PT, Pais P, Costa C, Manna S, Sa-Correia I, Teixeira MC 2017 The PathoYeastract database: an information system for the analysis of gene and genomic transcription regulation in pathogenic yeasts. Nucleic Acids Res 45:D597–D603. doi:10.1093/nar/gkw817. 27625390 PMC5210609

[42] Hopke A, Brown AJP, Hall RA, Wheeler RT 2018 Dynamic fungal cell wall architecture in stress adaptation and immune evasion. Trends Microbiol 26:284–295. doi:10.1016/j.tim.2018.01.007. 29452950 PMC5869159

[43] Shirtliff ME, Krom BP, Meijering RAM, Peters BM, Zhu J, Scheper MA, Harris ML, Jabra-Rizk MA 2009 Farnesol-induced apoptosis in *Candida albicans*. Antimicrob Agents Chemother 53:2392–2401. doi:10.1128/AAC.01551-08. 19364863 PMC2687256

[44] Spiering MJ, Moran GP, Chauvel M, MacCallum DM, Higgins J, Hokamp K, Yeomans T, d'Enfert C, Coleman DC, Sullivan DJ 2010 Comparative transcript profiling of *Candida albicans* and *Candida dubliniensis* identifies SFL2, a *C. albicans* gene required for virulence in a reconstituted epithelial infection model. Eukaryot Cell 9:251–265. doi:10.1128/EC.00291-09. 20023067 PMC2823005

[45] Harcus D, Nantel A, Marcil A, Rigby T, Whiteway M 2004 Transcription profiling of cyclic AMP signaling in *Candida albicans*. Mol Biol Cell 15:4490–4499. doi:10.1091/mbc.e04-02-0144. 15269278 PMC519143

[46] Tournu H, Luna-Tapia A, Peters BM, Palmer GE 2017 *In vivo* indicators of cytoplasmic. vacuolar, and extracellular pH using pHluorin2 in *Candida albicans*. mSphere 2:e00276-17. doi:10.1128/mSphere.00276-17. 28685162 PMC5497024

[47] Inglis DO, Arnaud MB, Binkley J, Shah P, Skrzypek MS, Wymore F, Binkley G, Miyasato SR, Simison M, Sherlock G 2012 The *Candida* genome database incorporates multiple *Candida* species: multispecies search and analysis tools with curated gene and protein information for *Candida albicans* and *Candida glabrata*. Nucleic Acids Res 40:D667–D674. doi:10.1093/nar/gkr945. 22064862 PMC3245171

[48] Sheehan G, Kavanagh K 2019 Proteomic analysis of the responses of *Candida albicans* during infection of *Galleria mellonella* larvae. J Fungi (Basel) 5:E7. doi:10.3390/jof5010007. 30641883 PMC6463115

