# Supplementary figures and images for: Remasking of Candida albicans β-Glucan in Response to Environmental pH Is Regulated by Quorum Sensing
Source: mBio. 2019 Oct 15;10(5):e02347-19. doi: 10.1128/mBio.02347-19 (PMC6794483; doi:10.1128/mBio.02347-19)

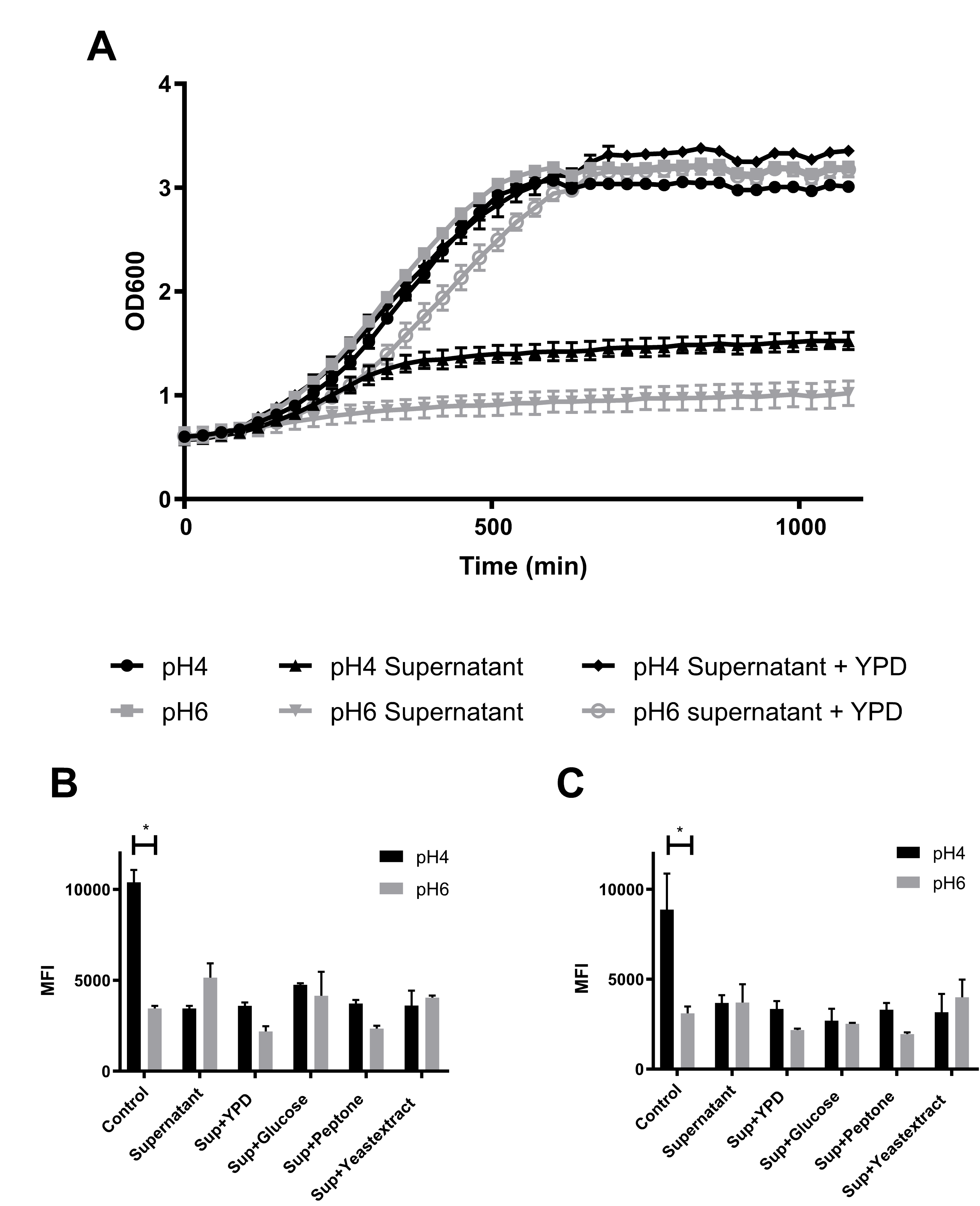

Supplement: FIG S3 [file mBio.02347-19-sf003.tif]

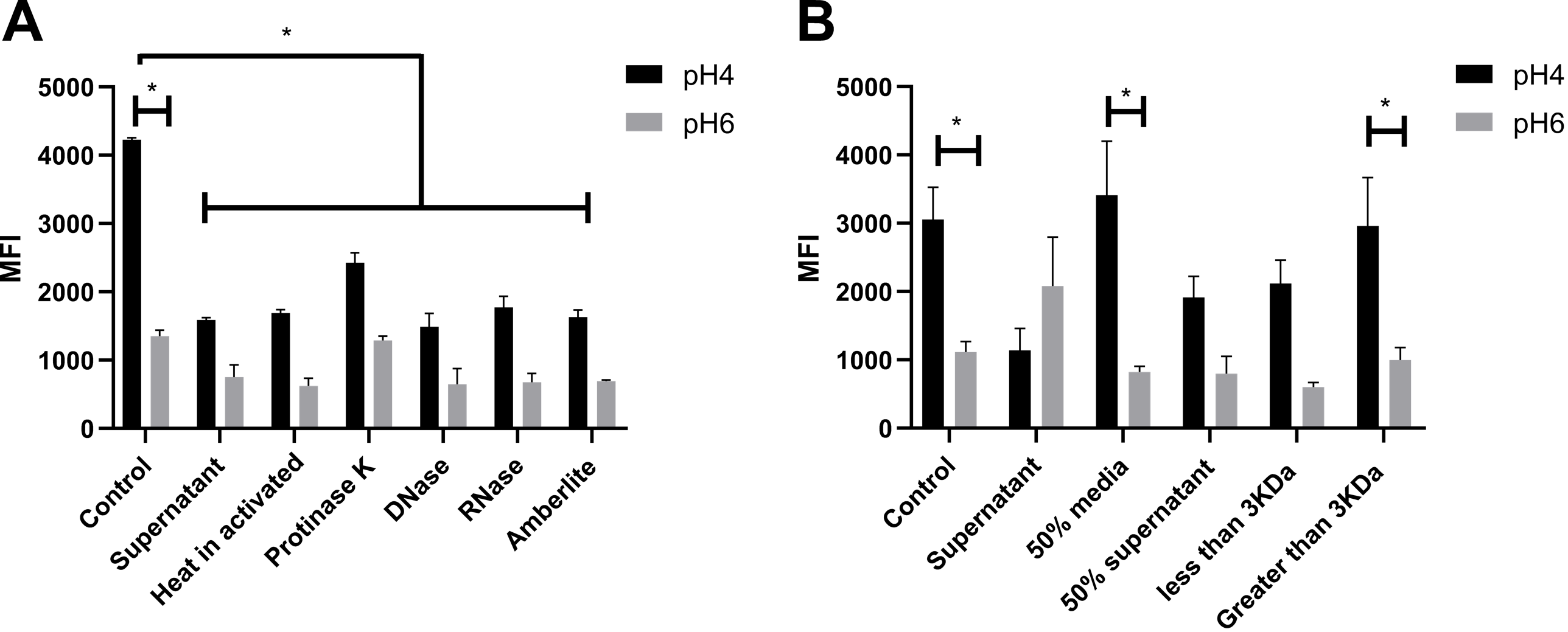

Supplement: FIG S4 [file mBio.02347-19-sf004.tif]
