# Supplementary material for: Remasking of Candida albicans β-Glucan in Response to Environmental pH Is Regulated by Quorum Sensing
Source: mBio. 2019 Oct 15;10(5):e02347-19. doi: 10.1128/mBio.02347-19 (PMC6794483; doi:10.1128/mBio.02347-19)
Supplement: TABLE S1 [file mBio.02347-19-st001.docx]

**Table S1: Strains used in this study.**

| Name | Organism | Genotype | Source |
| --- | --- | --- | --- |
| SC5314 | *C. albicans* |  |  |
| 7B | *C. albicans* | clinical isolate | Ramage G. |
| 10B | *C. albicans* | clinical isolate | Ramage G. |
| 15B | *C. albicans* | clinical isolate | Ramage G. |
| SN152 | *C. albicans* | MTLa/ α arg4Δ/arg4, leu2Δ/leu2, his1Δ/his1,URA3/ura3::λimm434, IRO1/iro1::λimm434, arg4::hisG/arg4::hisG his1::hisG/his1::hisG, leu2::hisG/leu2::hisG | (1) |
| *tpk1Δ* | *C. albicans* | MTLa/α arg4/arg4, leu2/leu2, his1/his1, ura3:: λimm434::URA3-IRO1/ura3:: λimm434, tpk1::HIS1/ tpk1::ARG4 | (1) |
| *tpk2Δ* | *C. albicans* | MTLa/α arg4/arg4, leu2/leu2, his1/his1, ura3:: λimm434::URA3-IRO1/ura3:: λimm434, tpk2::HIS1/ tpk2::ARG4 | (1) |
| *tpk1Δ tpk2Δ* | *C. albicans* | MTLa/α arg4/arg4, leu2/leu2, his1/his1,  ura3:: λimm434/ura3:: λimm434 iro1/iro1::  λimm434, tpk2::HIS1/ tpk2::ARG4,  tpk1::LEU2/ tpk1::FRT | (1) |
| CAI4+pSM2 | *C. albicans* | ura3 imm434/ura3::1 imm434, with pSM2 at *IRO1* locus | (2) |
| *cyr1Δ*+pSM2 | *C. albicans* | ura3 imm434/ura3::1 imm434 cdc35::hisG/cdc35::hisG, with pSM2 at *IRO1* locus | (2) |
| SN250 | *C. albicans* | *his1Δ/his1Δ, leu2Δ::C.dubliniensis HIS1 /leu2Δ::C.maltosa LEU2, arg4Δ /arg4Δ, URA3/ura3Δ::imm434, IRO1/iro1Δ::imm434* | (3) |
| *ifd6Δ* | *C. albicans* | *his1Δ/his1Δ, leu2Δ /leu2Δ, arg4Δ /arg4Δ, URA3/ura3Δ::imm434,RO1/iro1Δ::imm434, ifd6Δ::C.dubliniensisHIS1/ifd6Δ::C.maltosaLEU2* | (3) |
| *fet99Δ* | *C. albicans* | *his1Δ/his1Δ, leu2Δ /leu2Δ, arg4Δ /arg4Δ, URA3/ura3Δ::imm434,RO1/iro1Δ::imm434, fet99Δ::C.dubliniensisHIS1/fet99Δ::C.maltosaLEU2* | (3) |
| *hak1Δ* | *C. albicans* | *his1Δ/his1Δ, leu2Δ /leu2Δ, arg4Δ /arg4Δ, URA3/ura3Δ::imm434,RO1/iro1Δ::imm434, hak1Δ::C.dubliniensisHIS1/hak1Δ::C.maltosaLEU2* | (3) |
| *crz2Δ* | *C. albicans* | *his1Δ/his1Δ, leu2Δ /leu2Δ, arg4Δ /arg4Δ, URA3/ura3Δ::imm434,RO1/iro1Δ::imm434, crz2Δ::C.dubliniensisHIS1/crz2Δ::C.maltosaLEU2* | (4) |
| *dag7Δ* | *C. albicans* | *his1Δ/his1Δ, leu2Δ /leu2Δ, arg4Δ /arg4Δ, URA3/ura3Δ::imm434,RO1/iro1Δ::imm434, dag7Δ::C.dubliniensisHIS1/dag7Δ::C.maltosaLEU2* | (3) |
| *hgt6Δ* | *C. albicans* | *his1Δ/his1Δ, leu2Δ /leu2Δ, arg4Δ /arg4Δ, URA3/ura3Δ::imm434,RO1/iro1Δ::imm434, hgt6Δ::C.dubliniensisHIS1/hgt6Δ::C.maltosaLEU2* | (3) |
| *phr1Δ* | *C. albicans* | *his1Δ/his1Δ, leu2Δ /leu2Δ, arg4Δ /arg4Δ, URA3/ura3Δ::imm434,RO1/iro1Δ::imm434, phr1Δ::C.dubliniensisHIS1/phr1Δ::C.maltosaLEU2* | (3) |
| *fet31Δ* | *C. albicans* | *his1Δ/his1Δ, leu2Δ /leu2Δ, arg4Δ /arg4Δ, URA3/ura3Δ::imm434,RO1/iro1Δ::imm434, fet31Δ::C.dubliniensisHIS1/fet31Δ::C.maltosaLEU2* | (3) |
| *ihd1Δ* | *C. albicans* | *his1Δ/his1Δ, leu2Δ /leu2Δ, arg4Δ /arg4Δ, URA3/ura3Δ::imm434,RO1/iro1Δ::imm434, ihd1Δ::C.dubliniensisHIS1/ihd1Δ::C.maltosaLEU2* | (3) |
| *rim101Δ* | *C. albicans* | *his1Δ/his1Δ, leu2Δ /leu2Δ, arg4Δ /arg4Δ, URA3/ura3Δ::imm434,RO1/iro1Δ::imm434, rim101Δ::C.dubliniensisHIS1/rim101Δ::C.maltosaLEU2* | (4) |
| *efg1Δ* | *C. albicans* | *his1Δ/his1Δ, leu2Δ /leu2Δ, arg4Δ /arg4Δ, URA3/ura3Δ::imm434,RO1/iro1Δ::imm434, efg1Δ::C.dubliniensisHIS1/efg1Δ::C.maltosaLEU2* | (4) |
| *skn7Δ* | *C. albicans* | *his1Δ/his1Δ, leu2Δ /leu2Δ, arg4Δ /arg4Δ, URA3/ura3Δ::imm434,RO1/iro1Δ::imm434, skn7Δ::C.dubliniensisHIS1/skn7Δ::C.maltosaLEU2* | (4) |
| *tye7Δ* | *C. albicans* | *his1Δ/his1Δ, leu2Δ /leu2Δ, arg4Δ /arg4Δ, URA3/ura3Δ::imm434,RO1/iro1Δ::imm434, tye7Δ::C.dubliniensisHIS1/tye7Δ::C.maltosaLEU2* | (4) |
| *cas5Δ* | *C. albicans* | *his1Δ/his1Δ, leu2Δ /leu2Δ, arg4Δ /arg4Δ, URA3/ura3Δ::imm434,RO1/iro1Δ::imm434, cas5Δ::C.dubliniensisHIS1/cas5Δ::C.maltosaLEU2* | (4) |
| *ndt80Δ* | *C. albicans* | *his1Δ/his1Δ, leu2Δ /leu2Δ, arg4Δ /arg4Δ, URA3/ura3Δ::imm434,RO1/iro1Δ::imm434, ndt80Δ::C.dubliniensisHIS1/ndt80Δ::C.maltosaLEU2* | (4) |
| *pho4Δ* | *C. albicans* | *his1Δ/his1Δ, leu2Δ /leu2Δ, arg4Δ /arg4Δ, URA3/ura3Δ::imm434,RO1/iro1Δ::imm434, pho4Δ::C.dubliniensisHIS1/pho4Δ::C.maltosaLEU2* | (4) |
| JCH15448-1 | *C. auris* | clinical isolate |  |
| CAY676 | *C. tropicalis* | clinical isolate |  |
| WU284 | *C. dubliniensis* |  | (5) |
| 2001 | *C. glabrata* |  | (6) |
| AM13/0001 | *S. cerevisiae* | clinical isolate | MacCallum D. |
| CAI4 | *C. albicans* | *ura3*::imm434/*ura3*::imm434 *iro1/iro1*::imm434 | (7) |
| CAI4+pKE4-PHL2 | *C. albicans* | Like CAI4 but with pKE4-PHL2 at *URA3* locus | (8) |

**References**

1. Cao C, Wu M, Bing J, Tao L, Ding X, Liu X, Huang G. 2017. Global regulatory roles of the cAMP/PKA pathway revealed by phenotypic, transcriptomic and phosphoproteomic analyses in a null mutant of the PKA catalytic subunit in Candida albicans. Mol Microbiol 105:46-64.

2. Hall RA, De Sordi L, Maccallum DM, Topal H, Eaton R, Bloor JW, Robinson GK, Levin LR, Buck J, Wang Y, Gow NA, Steegborn C, Muhlschlegel FA. 2010. CO(2) acts as a signalling molecule in populations of the fungal pathogen Candida albicans. PLoS Pathog 6:e1001193.

3. Noble SM, French S, Kohn LA, Chen V, Johnson AD. 2010. Systematic screens of a Candida albicans homozygous deletion library decouple morphogenetic switching and pathogenicity. Nat Genet 42:590-8.

4. Homann OR, Dea J, Noble SM, Johnson AD. 2009. A Phenotypic Profile of the Candida albicans Regulatory Network. Plos Genetics 5.

5. Morschhauser J, Ruhnke M, Michel S, Hacker J. 1999. Identification of CARE-2-negative Candida albicans isolates as Candida dubliniensis. Mycoses 42:29-32.

6. Gerwien F, Safyan A, Wisgott S, Brunke S, Kasper L, Hube B. 2017. The Fungal Pathogen Candida glabrata Does Not Depend on Surface Ferric Reductases for Iron Acquisition. Front Microbiol 8:1055.

7. Fonzi WA, Irwin MY. 1993. Isogenic strain construction and gene mapping in Candida albicans. Genetics 134:717-28.

8. Tournu H, Luna-Tapia A, Peters BM, Palmer GE. 2017. In Vivo Indicators of Cytoplasmic, Vacuolar, and Extracellular pH Using pHluorin2 in Candida albicans. mSphere 2.

9. Cottier F, Raymond M, Kurzai O, Bolstad M, Leewattanapasuk W, Jimenez-Lopez C, Lorenz MC, Sanglard D, Vachova L, Pavelka N, Palkova Z, Muhlschlegel FA. 2012. The bZIP transcription factor Rca1p is a central regulator of a novel CO(2) sensing pathway in yeast. PLoS Pathog 8:e1002485.
