# Supplementary material for: Remasking of Candida albicans β-Glucan in Response to Environmental pH Is Regulated by Quorum Sensing
Source: mBio. 2019 Oct 15;10(5):e02347-19. doi: 10.1128/mBio.02347-19 (PMC6794483; doi:10.1128/mBio.02347-19)
Supplement: TABLE S2 [file mBio.02347-19-st002.docx]

**Table S2: Primers used in this study.**

| Name | Sequence | Reference |
| --- | --- | --- |
| ACT1-RT-F | CCTACGTGTACTTGTGCAAGGCAA | (9) |
| ACT1-RT-R | TAGTTGTGTGCACTGAGCGTCGAA | (9) |
| CHT2-RT-F | AATGTGTTGCCACTCCAGTT | (9) |
| CHT2-RT-R | CGGTGCATACAACAGTTTGA | (9) |
